# Information theory optimization of signals from small-angle scattering measurements

**DOI:** 10.1016/j.bpj.2025.06.031

**Published:** 2025-06-27

**Authors:** Robert P. Rambo, John A. Tainer

**Affiliations:** 1 Diamond Light Source Limited, Harwell Science and Innovation Campus, Didcot, United Kingdom; 2 Molecular Biophysics and Integrated Bioimaging, Lawrence Berkeley National Laboratory, Berkeley, California; 3 Department of Molecular and Cellular Oncology, The University of Texas MD Anderson Cancer Center, Houston, Texas

## Abstract

Small-angle X-ray scattering (SAXS) of particles in solution informs on the conformational states and assemblies of biological macromolecules (bioSAXS) outside of cryo- and solid-state conditions. In bioSAXS, the SAXS measurement under dilute conditions is resolution limited, and through an inverse Fourier transform, the measured SAXS intensities directly relate to the physical space occupied by the particles via the P(r)-distribution. Yet, this inverse transform of SAXS data has been historically cast as an ill-posed, ill-conditioned problem requiring an indirect approach. Here, we show that through the applications of matrix and information theories, the inverse transform of SAXS intensity data is a well-conditioned problem. The so-called ill-conditioning of the inverse problem is directly related to the Shannon number. By exploiting the oversampling enabled by modern detectors, a direct inverse Fourier transform of the SAXS data is possible, provided the recovered information does not exceed the Shannon number. The Shannon limit corresponds to the maximum number of significant singular values that can be recovered in a SAXS experiment, suggesting this relationship is a fundamental property of band-limited inverse integral transform problems. This correspondence reduces the complexity of the inverse problem to the Shannon limit and maximum dimension. We propose a hybrid scoring function using an information theory framework that assesses both the quality of the model-data fit as well as the quality of the recovered P(r)-distribution. The hybrid score utilizes the Akaike information criteria and Durbin-Watson statistic that considers parameter-model complexity, i.e., degrees of freedom, and the randomness of the model-data residuals. The described tests and findings extend the boundaries for bioSAXS by completing the information theory formalism initiated by Peter B. Moore to enable a quantitative measure of resolution in SAXS, robustly determine maximum dimension, and more precisely define the best parameter model appropriately representing the observed scattering data.

## Significance

The inverse transform problem in small-angle scattering (SAS) of dispersed particles in solution is considered an ill-posed, ill-conditioned problem. Using information theory, we show that the so-called Shannon number, $N_{s}$, of bandwidth-limited signals defines the boundary at which a problem transitions from well-conditioned to ill-conditioned. In this framework, $N_{s}$ represents the maximum number of orthogonal elements that are available to the SAS measurement and defines a fundamental relationship that determines the effective resolution of the recovered P(r)-distribution. We show that the inverse problem can be solved directly using a Shannon-limited approach. The Shannon limit establishes model complexity and, when incorporated into the Akaike information criteria, aids in selecting the most likely P(r)-distribution.

## Introduction

Small-angle X-ray scattering (SAXS) of particles in solution is an established technique for investigating the conformational and assembly states of biological macromolecules (bioSAXS) outside of cryo- and solid-state conditions (1). A bioSAXS measurement results from the scattering of billions of randomly oriented molecules, providing statistical power to single-particle, microscopic observations. Under sufficiently dilute conditions, the SAXS experiment represents a resolution-limited measurement of the particles’ thermodynamic solution-state. It is a measurement that directly informs on the physical space occupied by particles in solution and can be envisaged as an average, weighted by the thermodynamic ensemble (2).

A SAXS measurement consists of a set of intensities, *I*(*q*), defined within a scattering vector (*q*) range [*q*_*min*_, *q*_*max*_]. Here, the scattering vector, *q*, is $4\pi \cdot \sin\left(\theta \right)\cdot \lambda^{-1}$ in units of reciprocal Å, with θ as the half-scattering angle and λ as the X-ray wavelength in Å. The scattering vector range is a consequence of the instrument’s geometry that includes sample-to-detector distance, active detector area, beamstop size, and X-ray source properties (e.g., divergence and wavelength). The useable scattering vector range is often a subset of the instrument’s observable range, where the particle scattering intensity must be sufficiently greater than the parasitic scattering from the beamstop, slits, and sample cell windows to optimally define *q*_*min*_. At larger *q* values, the quality of the buffer matching (background subtraction) and particle concentration will be major factors that determine *I*(*q*_*max*_). The useable [*q*_*min*_, *q*_*max*_] range defines the set of intensities that faithfully describes the scattering of the particles under investigation with minimal contributions from parasitic scatter and poor buffer matching.

Importantly, the useable SAXS dataset will consist of *J* points within [*q*_*min*_, *q*_*max*_]. This number has steadily increased with improvements in detector technologies from a few hundred points using a charge-coupled device detector to over 2400 points with the latest generation photon-counting detectors. The increased number of points within a fixed [*q*_*min*_, *q*_*max*_] implies a greater oversampling of the underlying SAXS signal. If we consider a fixed [*q*_*min*_, *q*_*max*_] bioSAXS experiment that measures a 21 kDa particle at 1 mg/mL using a detector with 200 points vs. 2000 points, the information content obtained from each measurement will be the same assuming the same point focused camera geometry. However, fitting the same incorrect model, (e.g., PDB or specified parameter set) to both datasets will demonstrate that the dataset with the greater number of points will artificially drive down a scoring metric, such as chi-squared, $\chi^{2}$, thus contributing to a fundamental false positive problem in SAXS (3). This observation establishes a paradox in bioSAXS where improvements in detector technologies that decrease pixel sizes will improve model-data agreements regardless of the model when using scoring metrics that treat each individual *I*(*q*) as an independent observation.

A path to resolving this conflict was presented by Peter B. Moore (4) in 1980. He presented a formalism of SAXS based on information theory (5) that demonstrated that the number of independent data points, *N*_*s*_, that could be measured up to a given *q*_*max*_ can be estimated as $N_{s}=q_{\max}\cdot d_{\max}\cdot \pi^{-1}$. A particle with a *d*_*max*_ of 240 Å measured to a *q*_*max*_ of 0.4 Å^−1^ implies that ∼31 data points are required to fully capture the information within the scattering vector range measured at $q_{m}=m\cdot \pi \cdot d_{\max}^{-1}$, where m is a positive integer. Furthermore, Moore showed that each measured intensity, *I*(*q*_*j*_), is a linear combination of a fundamental set of intensities measured at *I*(*q*_*m*_) (see supporting material). This implies that modern SAXS datasets are highly redundant and correlated (3).

Using this information theory framework, we previously showed (6) that application of the Shannon-Hartley theorem (noisy-channel coding theory) (7) guarantees error-free recovery of the SAXS signal as long as the sampling frequency, i.e., the distance between successive *q* values, is within the Shannon-Hartley limit (see supporting material). However, this finding imposes a fundamental question: what exactly is the signal that is being sampled by SAXS? Based on the classical description of SAXS in Eq. 1, an intensity, *I*(*q*), is a sine-integral transform (see supporting material) of the paired-length, P(r), distribution function, suggesting the measured SAXS signal at the specified *q* value is a sampling of the P(r)-distribution at a resolution specified by $\pi \cdot q^{-1}$.

The integral transform is defined within the maximum dimension (*d*_*max*_) of the particle; however, we take *d*_*max*_ to be the largest dimension of the measured thermodynamic ensemble.

(1) $$I\left(q\right)=4\pi \cdot \int_{0}^{d_{\max}}P\left(r\right)\frac{\sin\left(qr\right)}{qr}dr$$

Building upon the above considerations, we demonstrate a correspondence between matrix and information theories that provides a complete theoretical framework to enable a direct inverse Fourier transform of SAXS data to accurately recover the P(r)-distribution. Until now, the inverse transform has been historically cast as an ill-posed, ill-conditioned (8,9) problem; however, we show that with modern instrumentation and the application of information theory, the problem is, in fact, not ill-posed or ill- conditioned. Our formalism is developed into an information criteria for the determination of the most likely maximum dimension $\left(d_{\max}\right)$, and a general method is proposed that determines the useable scattering range [*q*_*min*_, *q*_*max*_] from a SAXS measurement that addresses the quality of the background subtraction. This formalism provides a more intuitive understanding of resolution, and we expect these foundational concepts will provide enabling methods for ongoing and advanced X-ray scattering analyses.

## Materials and methods

BMV RNA and xylanase samples were manually purified using a KW 402.5 (Shodex, Tokyo, Japan) column in buffer containing 20 mM MOPS (pH 6.5), 50 mM KCl, and 7.6 mM MgCl_2_. Peak fractions were taken for SAXS at SIBYLS beamline 12.3.1 (Advanced Light Source, Berkeley, California). Bovine serum albumin (BSA) samples were prepared at beamline B21 (Diamond Light Source, Didcot, United Kingdom). 10 mg of BSA was diluted to 5 mg/mL in PBS buffer. For SEC-SAXS, a 45 *μ*L sample was injected into a KW-403 (Shodex) column at 160 *μ*L per min. SAXS data were collected at 2 s intervals for 32 min. In all cases, an X-ray wavelength of 1 Å was used during measurements.

SAXS datasets were reduced with DAWN (B21) and in-house software (SIBYLS). All datasets were processed using the program ScÅtter (https://github.com/rambor/scatterIV).

For the model selection search, each model is scored according to Eq. 10 and sorted. The lowest score of the set is then selected as the best model and assigned an initial probability of 1. For each additional model in the set, a probability is assigned based on how different the score, *s*, is from the best using Eq. 2.

The algorithms for the methods described above (see supporting material) are coded in the program ScÅtter (JAVA 1.8), available at https://bl1231.als.lbl.gov/scatter/, and the source code can be viewed at the GitHub repository (rambor/scatterIV) under src/Version4/InverseTransform.

(2) $$prob=e^{\frac{s_{best}-s_{i}}{2}}$$

Since we are interested in the most likely $d_{\max}$, which will be tested through multiple regularization weights, α, we sum the results from Eq. 2 at a constant $d_{\max}$ for each α to give an amplitude G. The scores are subsequently normalized across the entire set, and a Gaussian kernel density estimation (KDE) is performed using a window of 2.5. The KDE represents the final likelihood score in Fig. 6. For each $d_{\max}$ in the search space, we calculate the following KDE:

(3) $$likelihood\left(d_{j}\right)=\frac{1}{2\pi }\cdot \sum_{i=0}^{all\;d_{\max}}G_{i}\cdot e^{-\frac{{\left(d_{i}-d_{j}\right)}^{2}}{2}}\text{.}$$

## Results

The Debye equation (Eq. 1) relates the X-ray scattering intensity, I(q), to the P(r)-distribution (10). The relationship in Eq. 1 suggests that the P(r)-distribution can be recovered from the SAXS intensities through an inverse integral transform. Yet, in practice, the results of the inverse transform are often undesirable due to variances in I(q), the sampling frequency of the observed *q* range (Δq), and the choice of *r* values. It has been proposed that the inverse transform could be performed using a set of basis functions, such as cubic splines (8), sine function (4), or Chebyshev or Zernicke (11,12) polynomials, such that a least-squares minimization could be used to perform the inverse transform through an indirect method.

Alternatively, the relationship in Eq. 1 can be described as a matrix operation (Eq. 4)

(4) $$S\times \vec{p}=\vec{I}\left(q\right)\;with\;\vec{p}={\left[p_{1}\ldots p_{m}\right]}^{T}\text{,}$$

where the matrix, S, contains the elements of the sine transform and the vector $\vec{p}$ contains the set of unknown values of the P(r)-distribution (see supporting material). For a set of J intensities and M equally spaced points along the domain of the P(r)-distribution, S has the dimensions of J rows and M columns.

If the errors in the measurements or the number of elements in $\vec{p}$ is too large, the problem is ill-posed, and the matrix S is ill-conditioned (13), thereby preventing a sensible linear least-squares solution. In these cases, a solution can be found by imposing expectations (e.g., positivity or smoothness) upon $\vec{p}$ through a technique known as regularization (9,14).

(5) $$minimize:{\left\|S\times \vec{p}-\vec{I}\left(q\right)\right\|}^{2}+\alpha \cdot \Omega$$

Here, an objective function is defined that seeks to minimize the difference between the calculated, $S\times \vec{p}$, and observed, $\vec{I}\left(q\right)$, intensities. The objective is augmented with the regularization function, Ω, which embodies the expectation, and the influence of Ω is controlled by α(α≥0). Notably, as α tends to infinity, the elements of $\vec{p}$ will tend to zero. Therefore, a proper α value must be determined empirically (8,9,14).

The programs GNOM (9) and BayesApp (14) implement a smoothness expectation for recovering the P(r)-distribution from SAXS intensities. Smoothness can be estimated from the second derivative of the P(r)-distribution, and in the case of GNOM, Ω is simply the squared sum of the second derivative evaluated at the points defined in $\vec{p}$. The squared sum of a vector is a length metric that is strictly positive and commonly referred to as the L2-norm. Alternatively, the magnitude of the expectation vector could be evaluated as the sum of the absolute values, a metric known as the L1-norm. The L1-norm is statistically robust and seeks to find a set of parameters that is least complex. In this regard, regularization using the L1-norm can be considered the least biased estimate of $\vec{p}$ (15). Fundamentally, how many r values should be used to construct $\vec{p}$? If one uses an orthogonal basis set to represent the P(r)-distribution, then how many terms should be included in the expansion? An arbitrarily large number of points or terms leads to an ill-conditioned problem. We can determine the condition number of the problem by calculating the singular value decomposition (SVD) of the matrix S. Here, the Debye equation is rearranged as the Fourier sine transform as in (4), where the integral transform is first rearranged to remove the constant term from the right-hand side.

(6) $$q\cdot I\left(q\right)=4\pi \cdot \int_{0}^{d_{\max}}\frac{P\left(r\right)}{r}\;\sin\left(qr\right)\;dr$$

If we let H(q)=q·I(q) and $Q\left(r\right)=P\left(r\right)\cdot r^{-1}$, then the Debye equation is put into standard form, illustrating the Fourier sine integral transform relationship between intensity space, H(q), and real space, Q(r).

(7) $$H\left(q\right)=4\pi \cdot \int_{0}^{d_{\max}}Q\left(r\right)\cdot \sin\left(qr\right)\;dr$$

The M unknowns are embodied by Q(r). Each term of S is, therefore, $\sin\left(q_{j}\cdot r_{m}\right)$, where j indexes over the observed [*q*_*min*_, *q*_*max*_] and *m* indexes over an arbitrary number of equally spaced r values within [0, *d*_*max*_]. From Eq. 4, the unknown terms in $\vec{p}$ could be recovered if S was invertible. It can be expected that S will not be a square matrix, i.e., M≠J, and likewise not invertible. For nonsquare matrices, a pseudo-inverse, $S^{+}$, can be calculated based on an SVD factorization of the S matrix.

The SVD of S will produce K singular values, $\sigma_{k}$, where K=min(J,M). Singular values are arranged from largest to smallest, and $S^{+}$ can be calculated by setting all insignificant singular values to zero. This effectively reduces the matrix to a set of linearly independent columns. A judgment must be made as to when a $\sigma_{k}$ is insignificant. We can quantify this by noting that the condition number of a matrix is the ratio of the first singular value, $\sigma_{1}$, to the last. A well-conditioned problem will have a low condition number, κ, ideally equal to unity. By plotting the ratio of $\sigma_{1}$ to all subsequent singular values in the set, we can demonstrate how the conditioning of the matrix evolves as the number of unknowns increase through the size of $\vec{p}$.

**Figure 1:**
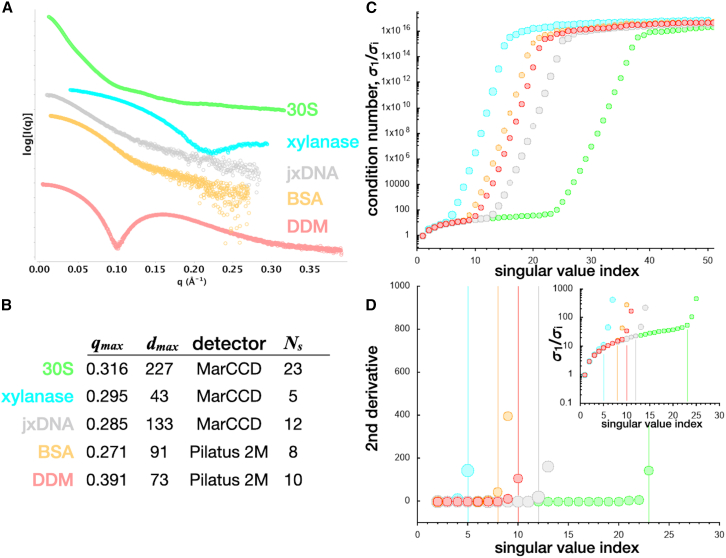
SAXS datasets of particles with varying size, shape, and composition. (*A*) Experimental datasets for the *Sulfolobus solfataricus* 30S ribosomal subunit (*green*), *B. subtilis* xylanase (*cyan*), 25 base-paired double-stranded DNA with an 11 nt overhang (*gray*), bovine serum albumin (BSA; *orange*), and n-dodecyl-beta-D-maltopyranoside (DDM) micelle (*red*). 30S, xylanase and jxDNA samples are size-exclusion-purified samples collected as a batch-mode SAXS experiment (16,17). BSA and DDM were collected as SEC-SAXS experiments in PBS buffer. (*B*) SAXS datasets were collected on either a charge-coupled device detector (18) or a silicon photon-counting detector (Pilatus 2M) with *Δ*q spacings of 0.000608 and 0.0002749 Å^−1^, respectively. Each dataset contains *N* data points with different ${\left[q,d\right]}_{\max}$ and different Shannon numbers, $N_{s}$. (*C*) For each [*q, d*]_*max*_, singular value decompositions were performed on S matrices with dimensions of N rows and 11 $\times N_{s}$ columns. For each S matrix, condition number plots were constructed by plotting the ratio of the i^th^ singular value to the first singular value. Insignificant singular values will drive successive condition numbers several orders of magnitude (note, *y* axis is log_10_). (*D*) Second derivative plot of the starting data in (*C*) shows condition number rate of change for each dataset in (*A*). All datasets illustrate a second derivative that has an initial constant region followed by an abrupt increase. The Shannon number for each dataset in (*B*) is indicated by the corresponding vertical line. The inset is the condition number plot of the subset of the data in (*D*). The DDM dataset was kindly provided by Mark Tully, ESRF (Grenoble, France).

Using several SAXS datasets of particles with varying $q_{\max}$ and $d_{\max}$ values (Fig. 1 *A* and *B*), the S matrix was calculated for an arbitrary number of r values, and an SVD was performed to determine the singular values for each $q_{\max}$, $d_{\max}$ pair (referred to as ${\left[q,d\right]}_{\max}$). Plotting the ratio of the singular values, as described above, shows a general trend where there exists a region of small condition numbers that abruptly increase (Fig. 1 *C*). The magnificent increase to infinity for each S matrix illustrates the ill-conditioned behavior for each combination of $q_{\max}$ and $d_{\max}$. The abrupt transition demarcates the boundary where all subsequent singular values can be considered insignificant, as the division by an exceedingly small number drives the ratio to infinity. Interestingly, this boundary is coincident with $N_{s}$, the Shannon number derived by Moore, and suggests $N_{s}$ can be used to determine the number of significant singular values for a given ${\left[q,d\right]}_{\max}$.

**Figure 2:**
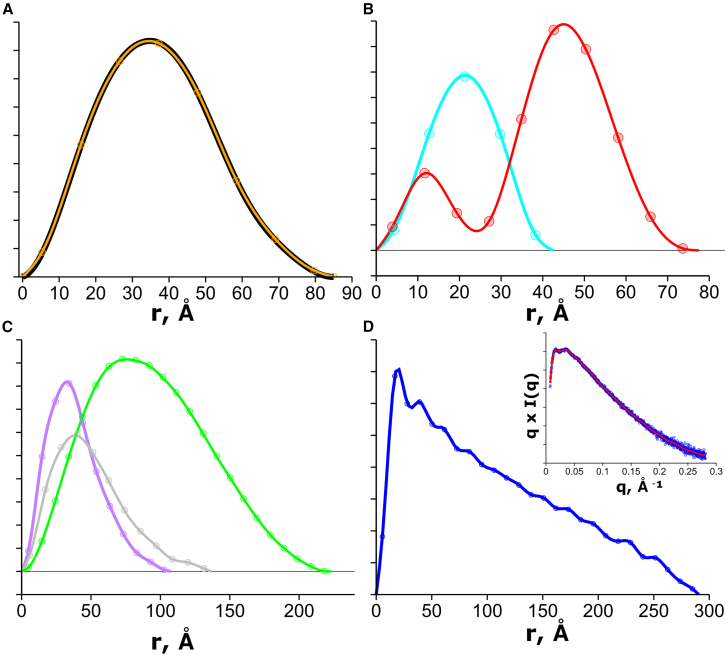
Recovery of P(r)-distributions using the Shannon-limited, pseudo-inverse (SPI) method for data in Fig. 1. (*A*) Transformation of the BSA data using GNOM (*black*) (9) and SPI (*orange*). Orange curve is the spline interpolated curve based on the SPI-determined points (*circles*). (*B*) Transformation of xylanase (*cyan*) and DDM (*red*). (*C*) SAXS data of nucleic acid proteins from Fig. 1 with P4-P6 RNA domain (*purple*). (*D*) SPI method applied to SAXS data (*inset*, plotted as q×I(q) versus q) of SEC-SAXS data of KASH5 coiled-coil protein. KASH5 data were kindly provided by Owen Davies, New Castle University. The *y* axis is in relative units, and distributions in (*B*) and (*C*) are scaled arbitrarily for presentation. Smoothed curves for each SPI inversion were performed with cubic splines in gnuplot.

If the S matrix is constructed using only $N_{s}$ columns, then the number of unknowns will be restricted to the number of significant eignevalues providing for a well-conditioned problem. Since the integral transforms in Eq. 7 are only defined between 0 and $d_{\max}$, there will be a set of r values separated by a Δr of $d_{\max}/N_{s}$ for each of the $\sin\left(q_{j}\cdot r_{m}\right)$ terms in the S matrix. Here, the P(r)-distribution is conceptualized as a histogram that is divided into $N_{s}$ bins, where each bin has equal bin widths of Δr. The p vector is therefore the height of each bin. Fig. 2 shows results for several SAXS datasets that are solved for the corresponding $\vec{p}$ using the pseudo-inverse, $S^{+}$.

Monomeric BSA with a [0.277174, 85]_max_ corresponds to an $N_{s}$ of eight, which implies an S matrix of eight columns and a $\vec{p}$ with eight unknowns. The direct recovery of the P(r) -distribution shows a $\vec{p}$ consistent with a distribution (Fig. 2 *A*). In fact, overlaying the components of $\vec{p}$ onto an L2-regularized indirect inverse transform that used 246 points shows an ideal coincidence (Fig. 2 *A*). The spline interpolation based on $\vec{p}$ shows a nearly perfect overlay with the L2-regularized inverse transform (Fig. 2 *A*), suggesting that the Shannon-limited, pseudo-inverse (SPI) method recovers the same information as the L2-regularization method.

The SPI method was applied to additional SAXS datasets (Fig. 2) of particles with varying aspect ratios and [*q, d*]_*max*_ pairs. The globular particles xylanase and BSA produced the expected bell-shaped distribution, with xylanase requiring only five independent Shannon points to represent 496 reciprocal-space data points. We also applied our method to mixed-phase particles, such as a n-dodecyl-beta-D-maltopyranoside (DDM) micelle and the 30S ribosomal subunit. For the DDM micelle, the recovered $\vec{p}$ with 10 $N_{s}$ displays the classic bimodal distribution of detergent micelles, whereas the 30S subunit at a $d_{\max}$ of 227 Å requires $N_{s}$ = 23 points to describe the large, asymmetric particle. Finally, we examined three highly asymmetric particles, as the SPI method should be insensitive to particle shapes. The P4-P6 RNA domain, a 25 base-paired (bp) double-stranded (ds) DNA with an 11-nt overhang, and a coiled-coil protein domain with a $d_{\max}$ larger than the 30S subunit were examined. In all cases, the recovered $\vec{p}$ produced a smooth distribution; however, the coiled-coil SAXS dataset shows subtle oscillatory features that could be real or an aliasing artifact.

Oscillatory features can result from special conditions between the interpolating polynomial and the underlying equispaced points (Runge’s phenomenon (19)). It must be emphasized that the equispaced points from the SPI method represent the actual resolution-limited, real-space data from the SAXS measurement, whereas the oscillation is a visual artifact. As the number of real-space points increases or decreases, oscillatory features from special conditions will vary, so a completely smooth, oscillation-free P(r)-distribution should not be a necessary condition for an acceptable P(r)-distribution. This can be best appreciated by examining $q_{\max}$-dependent changes in the atomistic P(r)-distribution calculated from a 137 bp dsDNA model (Fig. S1 *A*). At a $q_{\max}$ of 0.302 Å^−1^, there are 14 $N_{s}$ points that provide a relatively coarse description of a skewed distribution, and as $q_{\max}$ increases, the number of bins that define the P(r)-distribution will increase, producing a finer description of the distribution. More importantly, at a $q_{\max}$ of 0.302 Å^−1^, the bin width matches the pitch (10.4 Å) of a B-form dsDNA helix, and we see at this resolution that the P(r)-distribution is exceptionally smooth. However, as the bin width is decreased (akin to increasing the $N_{s}$), the distribution develops oscillatory features that appear to be most severe at even-number increments of 0.302 Å^−1^ and least at odd-number increments. The oscillation is due to an undulation between successive bins, and since the bin heights are calculated directly from the structure, the oscillation is entirely a consequence of the bin width and does not represent an unstable solution of the inverse transform.

**Figure 3:**
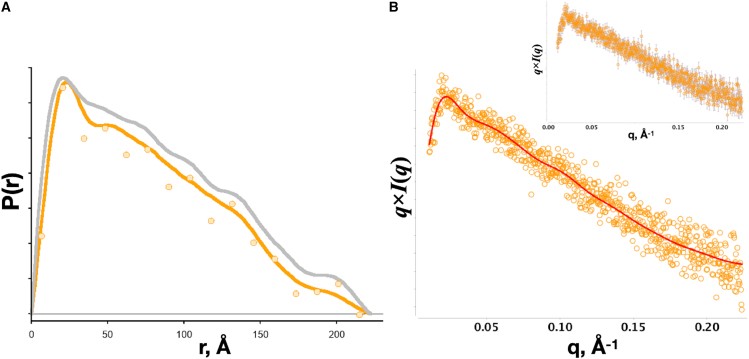
Real-space transformation of a high-noise SAXS dataset from a high-aspect-ratio particle (the coiled-coil protein SYP1 [pH 8], (20)). (*A*) P(r)-distribution recovered using inverse transform methods GNOM (*gray line*), SPI (*orange circle*), and Moore (*orange line*) with smoothness regularization as in GNOM. Using an excessive number of points (GNOM default 256 points) to describe the distribution has the effect of filling in features, as smoothness minimizes differences between neighboring points (see *valley* near 40 Å). The *y* axis plots the distribution in relative units. The data were kindly provided by Owen Davies, Institute Cell and Molecular Biology Newcastle University, United Kingdom. (*B*) SAXS dataset transformed as q×I(q) with the fit from the Moore method in (*A*) (*red line*). The inset shows the same data with error bars.

Regularization is a widely used tool for performing the inverse transform of SAXS data (8,9,14). It imposes an expectation on $\vec{p}$, and in the case of smoothness, the regularization attempts to minimize the differences between neighboring values in the P(r)-distribution. Yet, as discussed above (Fig. S1 *A*), such an expectation may unduly attenuate real features in a distribution as smoothness becomes an overvalued expectation, i.e., large α. Nevertheless, regularization has been essential in transforming smeared, noisy, and undersampled SAXS data. Today’s SAXS measurements will mainly suffer from noise, particularly as measurements are made at low particle concentrations, and regularization may be critical to a successful inverse transform. The determination of $S^{+}$ via SVD does not consider noise in the dataset. Fig. 3 shows the application of the SPI method on SAXS data with a large variance between successive data points (Fig. 3 *B*).

The SPI method fails to produce a solution that is smooth, as the oscillations persist through several choices of [*q, d*]_*max*_. Here, regularization will be critical to finding an appropriate solution. However, there will be variations in the solution depending on how the regularized inverse transform is performed (Figs. 3 *A* and S2), i.e., the size of $\vec{p}$ and the nature of the regularization. The goal is to recover the real-space information that is least biased.

The condition number analysis (Fig. 1) suggests that $N_{s}$ determines the maximum number of linearly independent columns of the S matrix. Columns that are linearly independent are orthogonal, which implies that the P(r)-distribution can be represented as an expanded series of orthogonal polynomials limited by the Shannon number, e.g., Legendre, Laguerre, Jacobi, or Hermite. In fact, the orthogonal series expansion is the basis for the method proposed by Moore (4), which uses a Fourier sine series (orthogonal trigonometry functions). In Glatter’s indirect Fourier transform method, the distribution is approximated as a set of nonorthogonal cubic B splines requiring smoothness regularization.

Since the P(r)-distribution is nonzero within a defined domain [0, $d_{\max}$], we can demonstrate the general application of orthogonal polynomial series expansion in inverse transforms using Legendre polynomials of degree *m,* $L_{m}$ (see supporting material), to represent the unknown P(r)-distribution. Legendre polynomial density approximations are inherently smooth and provide a well-established technique that makes no assumptions on the shape of the distribution (21).

(8) $$\int_{0}^{d_{\max}}P\left(r\right)\cdot \frac{\sin\left(qr\right)}{qr}\;dr\approx \int_{0}^{d_{\max}}\frac{\sin\left(qr\right)}{qr}\cdot \sum_{m=0}^{N_{s}}\lambda_{m}\cdot L_{m}\left(\frac{2r-d_{\max}}{d_{\max}}\right)\;dr$$

**Figure 4:**
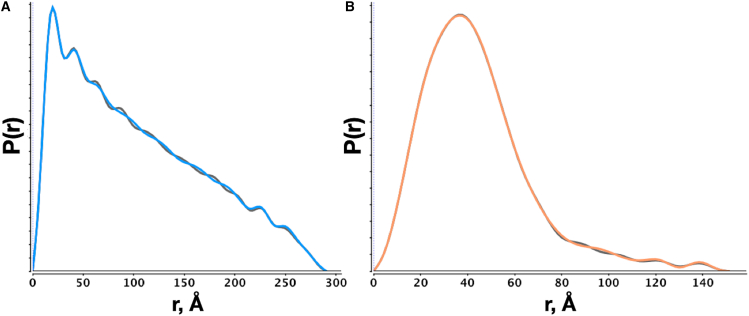
Indirect Fourier transforms (IFTs) using an orthogonal series expansion without regularization (α=0) (*A*) IFT of coiled-coil data in Fig. 1*D* using Legendre (*blue*) and SPI (*dark gray*) methods. (*B*) IFT of unpurified BSA using Legendre (*orange*) and SPI (*dark gray*) methods. $d_{\max}$ was fixed to the same value for each method in the respective datasets. Smoothed curves for each SPI inversion performed with cubic splines in gnuplot.

In this approach, each element in the S matrix will be a product sum of the sine term and a Legendre polynomial, whereas $\vec{p}$ contains the set of unknowns, $\lambda_{i}$, that can be determined through least squares (see supporting material). To test the robustness of the $N_{s}$-limited polynomial expansion fitting of SAXS data, we examined the coiled-coil data in Fig. 1 *D* and a dataset of unpurified BSA in PBS buffer (Fig. 4). The coiled-coil protein has a high aspect ratio with a cross-sectional diameter of 12 Å and a length of nearly 300 Å, whereas the BSA dataset will be a mixture of monomers and dimers with some higher-order oligomeric species, suggesting that the solution-state ensemble will consist of particles of varying $d_{\max}$ values. In comparison to the SPI method, the Legendre polynomial expansion method resulted in smoother distributions that are nearly identical at the extremes but differ within the medium-range distances for both datasets (Fig. 4). Furthermore, the $N_{s}$-limited polynomial expansion was tested using the Moore method (Fig. S2). The recovered Fourier sine series and Legendre-based distributions shows substantial agreements, though there are subtle differences between the two distributions that reflect the type of basis function used in the expansion.

Increasing the number of expansion terms beyond $N_{s}$ introduces instabilities or artifacts in the recovered distribution as the inverse transform problem becomes ill-conditioned. For both the Moore and Legendre-based methods, instabilities will contribute to oscillations and spurious features in the recovered distribution (Figs. S3 and S4). For a given [*q, d*]_*max*_, profiling the condition number (Fig. 1) shows how sensitive the S matrix will be to the number of unknowns, M. By restricting the matrix inversion or orthogonal series expansion to $N_{s}$, we maximize the information recovered while minimizing artifacts introduced from regularization or ill-conditioning.

The Shannon number is a quantitative measure that informs on the number of independent columns of the S matrix or terms used in an orthogonal series expansion. It represents the maximum number of nondegenerate elements of information contained within the solution-state SAXS measurement defined by [*q, d*]_*max*_. Practically, both $q_{\max}$ and $d_{\max}$ are unknowns in a SAXS measurement, as the former is not solely determined by the physical limitations of the detector. The largest scattering vector that contains useable intensity information, $I\left(q_{\max}\right)$, cannot be determined simply by a signal/noise ratio, $I\left(q_{i}\right)/\sigma_{i}$, as the Shannon-Hartley theorem stipulates that the ability to recover a signal relies on both the magnitude of $I\left(q_{i}\right)/\sigma_{i}$ and the sampling frequency, Δq. Likewise, assessing the quality of the inverse transform using a chi-squared metric alone has proven not to be reliable for choosing the appropriate $d_{\max}$ due to overfitting (4,6,9,14). The assessment must consider both the quality of the model-data agreement and the quality of the putative P(r)-distribution.

**Figure 5:**
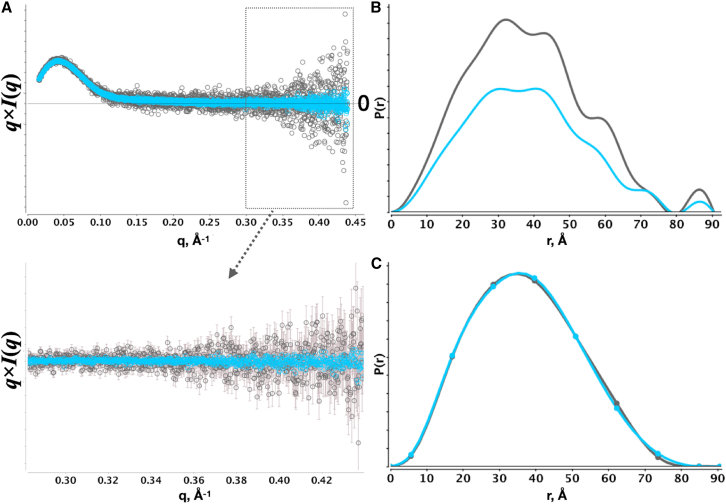
Monomeric BSA from SEC-SAXS of dilute BSA in PBS buffer. (*A*) Average of ∼20 frames across a single elution peak (*dark gray circles*) corresponding to ∼0.26 mg/mL versus an average of 18 independent, identical SEC-SAXS runs of monomeric BSA at the same ∼0.26 mg/mL concentration. The bottom left image (*arrow*) is subset of data plotted above with errors. (*B*) L1-norm regularized SPI method for both datasets in (*A*). (*C*) Regularized SPI method with constant background term as in (*B*). For both (*B*) and (*C*), IFT was achieved with α=0.007 and a $d_{\max}$ of 90.5 Å. Smoothed curves for each SPI inversion were performed with cubic splines in gnuplot.

In practice, determining both $q_{\max}$ and $d_{\max}$ is an iterative process where the recovered P(r)-distribution is visually assessed for smoothness, nonnegativity, and a shallow finish. If $q_{\max}$ is too large, the dataset may include intensities that carry no information. Here, the buffer-subtracted intensities at high q will be randomly distributed around some average value, b. Ideally, the average should be zero, but for imperfect buffer-subtracted samples, b will be a nonzero constant that the SAXS intensities at high q will be distributed around. The effect of b can be readily illustrated using SEC-SAXS data of BSA collected under very dilute conditions (0.26 mg/mL). The sample is ideally buffer matched; however, the intensities become distributed near zero (Fig. 5 *A*) beyond moderate scattering vectors (q > 0.2). Performing the inverse transform of the SAXS data without a constant background term resulted in a bulging of the distribution near $d_{\max}$ (Fig. 5 *B*) using the Legendre, Moore, and SPI methods. This bulging persisted with a SAXS dataset of the same sample that was averaged over 17 independent SEC-SAXS runs, suggesting that the low signal/noise ratio is not responsible for the bulge. In addition, attenuating the bulge through a large α introduced artifacts in the distribution or led to an overestimated $d_{\max}$. For both SAXS datasets, performing the inverse transform with a constant background term in the S matrix provided for an ideal P(r)-distribution (Figs. 5 *C* and S5). Notably, regularization was critical to the successful inverse transform, suggesting that for weakly measured signals near or within the background, the regularization with a constant b term can optimally recover a P(r)-distribution. The contribution of b becomes significant under very dilute conditions as the subtracted intensities are within the background. The *q* value at which the intensities become evenly and randomly distributed about b suggests a $q_{\max}$ for the SAXS dataset.

### Model selection

A given [*q, d*]_*max*_ and α collectively define the parameters of a particular inverse transform model (e.g., Moore, Glatter, or SPI). Therefore, finding the “best” parameter-model is a search in a multidimensional parameter space where each parameter-model must be evaluated not only for the quality of the fit to the experimental intensities but also for the quality of the recovered P(r)-distribution, Ψ. Such a search represents a selection within a constrained search space, and since the complexity of the parameter-model will scale with $N_{s}$, the selection of the best parameter-model must weigh the risk of overfitting. This can be readily achieved by incorporating the Akaike information criterion (AIC) (22) into an integrated score. The AIC has been successfully applied to ensemble modeling of SAXS data using atomistic models (23). Here, we use a modification of the criterion $\left(AIC_{c}\right)$ that includes corrections for small sample sizes that are imposed by $\chi^{2}$*-free* (6):

(9) $$AIC_{c}=2\cdot N_{p}+N_{s}\cdot \chi_{free}^{2}+\frac{2d_{f}\cdot \left(N_{p}+1\right)}{T-N_{p}-1}\text{,}$$

where $N_{p}$ is the total parameters used in the fit and T is the sample size used to calculate $\chi^{2}$-free. The $N_{p}$ is taken as $N_{s}$ plus the three that includes assumptions on α, $d_{\max}$, and that the residuals between the model-data agreement are Gaussian. To assess the model-data agreement, we will use an alternate to $\chi^{2}$ by noting that an ideal agreement is characterized by a random distribution of the residuals. We can quantitate this randomness via the Durbin-Watson statistic (D_W_), which examines the auto-correlation of the residuals with a lag of 1 (24). D_W_ is bounded between 0 and 4 and will be 2 for perfectly random residuals. Thus, we can construct a metric as the absolute value of (2 − D_W_). For Ψ, we require a function that quantitates the finish near $d_{\max}$ and the overall smoothness of the P(r)-distribution and penalizes for negative values (see supporting material). Our overall score is given by Eq. 10,

(10) $$score=0.1\cdot AIC_{c}+4\cdot \left|2-D_{W}\right|+\Psi \text{,}$$

with the scaling factors, 0.1 and 4, empirically determined. At fixed $q_{\max}$, our scoring function can be applied to a set of models that vary by $d_{\max}$ and α, where the minimum score identifies the best parameter-model of the set. However, we can collectively combine the scores into a likelihood function that illustrates the region of the most likely $d_{\max}$ of the dataset (see supporting material).

**Figure 6:**
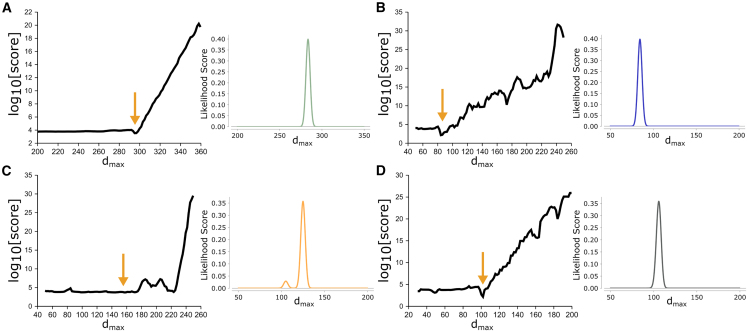
Scoring of P(r)-distributions in an $\alpha ,d_{\max}$ search. (*A*) Coiled-coil protein from Fig. 2*D*. (*B*) SEC-SAXS-purified BSA from Fig. 2*A*. (*C*) Unpurified BSA from Fig. 4*D*. (*D*) Mixture of BMV TLS RNA (*d*_*max*_ of 108 Å) and xylanase (*d*_*max*_ of 43 Å). Note the asymmetry in the likelihood peak or presence of multiple peaks for samples that are mixtures. For each image, the righthand plot illustrates the likelihood score calculated from the set of scores for each search. All searches were performed with the Moore method using smoothness regularization, and all *x* axes are in units of Å. The *d*_*max*_ search range is given by the *x* axis for each image. BMV TLS RNA was kindly provided by Jeff Kieft, University Colorado School of Medicine, Boulder, Colorado.

Using SEC-SAXS data of a coil-coiled protein and BSA (Fig. 6 *A* and *B*), the scoring function shows a clear minimum at the expected $d_{\max}$. The scoring function is relatively flat before an optimal $d_{\max}$, as the score is largely determined by $AIC_{c}$. After $d_{\max}$, the scoring function increases rapidly as the penalty contributions from oscillations, the presence of negative values, and the finish near $d_{\max}$ become more substantial. The SEC-SAXS samples are structurally homogeneous and monodispersed, implying that each dataset can be described by a single $d_{\max}$ value. For more challenging samples, such as the unpurified BSA dataset, the overall score does not show a clear minimum; in fact, the likelihood function shows multiple peaks (Fig. 6 *C*), implying an ambiguity in assigning $d_{\max}$. The sample is dominated by monomers but also contains dimers and additional oligomers, suggesting that $d_{\max}$ will not be due to a single structural species. The ambiguity in determining a definitive $d_{\max}$ reflects the poor quality of the sample, and in this regard, the likelihood function illustrates a single, symmetric peak for well-defined, monodispersed samples versus multiple or broad peaks (Fig. 6 *C* and *D*) for poly-dispersed systems. The search method and use of our score in a likelihood function were further tested on nonglobular particles that include a DDM micelle, 25 bp dsDNA with a 10-nt single-stranded overhang (17), 50 bp dsDNA, unfolded SAM riboswitch in EDTA (25), amphipol-stabilized G-protein-coupled receptor (26), and BMV TLS RNA (27) (see Figs. S6 and S7).

## Discussion

Since the foundational contributions of Glatter and Moore in the 1970s, the modern advances in X-ray focusing optics, monochromators, and detector technologies now provide highly oversampled and focused SAXS measurements that are free of smearing artifacts (10,28,29,30). Likewise, the availability of SEC-SAXS at synchrotron facilities (31) has enabled ideal buffer (background) matching for producing subtracted SAXS signals of biological particles. This convergence of sample preparation and instrumentation now provides for reliable and routine measurements of the SAXS signal that can be exploited to overcome previous limitations.

Using the information theory framework, we see that the Shannon number, $N_{s}$, corresponds to the boundary between a well- and an ill-conditioned problem. For a given [*q, d*]_*max*_, this boundary position determines the maximum number of orthogonal components required to fully describe the inverse SAXS problem. Any additional components makes the problem ill-conditioned, thereby requiring some form of regularization (e.g., nonnegativity or smoothness) to solve for the unknowns. A nonnegativity regularization reduces the size of the search space, whereas a smoothness regularization imposes correlations between P(r) values to reduce the variation between neighboring values. Alternatively, an orthogonality regularization could be performed to optimally maintain independence between the unknowns. Nevertheless, regularization is a balance between maintaining strict independence of the unknowns in $\vec{p}$ and the orthogonality of the columns of the S matrix. This balance can best be achieved by restricting the complexity of the problem to $N_{s}$ and utilizing a low α in regularization. The goal of the inverse transform is to recover the real-space information that is least biased and free of algorithm or regularization artifacts.

The SPI method solves for a $\vec{p}$ with $N_{s}$ terms by dividing the P(r)-distribution into bins of equal width, $b_{w}$, given by $d_{\max}/N_{s}$. Using the relationship described by Moore, we see that $b_{w}$ is fundamentally determined by $q_{\max}$ (Eq. 11). Regardless of the size of the particle, the effective resolution of the P(r)-distribution is essentially $b_{w}$, meaning that no distance can be resolved to better than $b_{w}$.

(11) $$b_{w}:=\frac{\pi }{q_{\max}}=\frac{d_{\max}}{N_{s}}$$

Equation 11 also implies that a higher-resolution measurement, i.e., increasing $q_{\max}$, will increase $N_{s}$, thereby reducing the width of the bin used to define the P(r)-distribution. A smaller $b_{w}$ increases the level of detail in the P(r)-distribution, producing a higher-information-content SAXS measurement. This relationship is invariant to how the inverse transform is performed, e.g., SPI, Legendre, Moore, GNOM, or GIFT. It is only through increasing $q_{\max}$ that the information content or details of the P(r)-distribution can be increased.

Inverse integral transform problems using data with typical measurement noise will not produce a solution that is uniquely determined with high confidence when using a $\chi^{2}$-like metric. Various methods have been proposed, including Bayesian-based (14) and hybrid scores (9), to assess model-data agreements similar to the approach presented above. Determining a $d_{\max}$ from a bioSAXS experiment is essentially accepting the $d_{\max}$ that gives the most acceptable features in the distribution. Proposed nearly 30 years ago, this perception (9) of the best answer was developed into a score or perceptual criteria that quantified a leading SAXS expert’s experience and knowledge into a useable score for the inexperienced, novice user. Using $AIC_{c}$, our approach improves upon this type of approach by further considering the complexity of the inverse transform model. The $AIC_{c}$ tries to find the least complex answer that is balanced by perception akin to Occam’s razor. Our proposed score (Eq. 10) does have its limitations, as it penalizes for negative P(r) values. There may be instances in contrast variation or anomalous SAXS measurements that support negative values in the P(r)-distribution, and in these cases, it would be prudent to consider a different method for assessing Ψ. Equation 10 is modular and readily allows adjustments to Ψ to compensate for atypical SAS experiments.

## Conclusion

The *P*(*r*)-distribution is the most reliable representation of a bioSAXS measurement, as each point in the distribution is determined from the entire set of observed SAXS intensities. Preserving the fidelity of the information through the inverse transform is paramount in comparative studies, detecting conformations changes or any modeling based on the *P*(*r*)-distribution. The information is resolution limited, and the requirements to transform the data, i.e., determination of $d_{\max}$ and $q_{\max}$, coincide with the Shannon limit and the boundary at which the inverse problem becomes ill-conditioned. Crossing this boundary imposes regularization restraints and risks the fidelity of the SAXS information. We present a quantitative, information-theory-based method to identify parameter models in best agreement with observed scattering data while avoiding overfitting. The reported method objectively determines the critical maximum dimension value and provides a quantitative measure of resolution. Inverse transforms will be sensitive not only to how the data are transformed (e.g., Moore versus SPI method) but also to the requirement for regularization. This sensitivity may introduce artifacts (Fig. 4) in the recovered distribution, and it must be emphasized that comparative studies (i.e., apo versus bound states) be made using the same inverse transform method. We envisage future developments that target the *P*(*r*)-distribution in atomistic refinements as well as ab initio modeling, which unduly requires a *P*(*r*)-distribution that is least biased. BioSAXS has an unrealized potential to provide comprehensive measurements of dynamic macromolecular conformations and assemblies in solution that are directly relevant to determining biological structure-function relationships (1,32,33). SAXS routinely extends and complements the structural knowledge obtained from crystallography (34), NMR (35,36,37), and now, cryoelectron microscopy (38), where molecular dynamics-coupled SAXS modeling can discover new biological insights and assist in computational protein design (39,40). In fact, cryoelectron microscopy structures of molecular machines, such as the transcription preinitiation complex, suggest that large, macromolecular motions are fundamental processes that are well suited for the SAXS analyses described here (41).

## Acknowledgments

We thank N. Cowieson, K. Inoue, and Nikul Khunti of the Diamond Light Source (Didcot, United Kingdom), J. Doutch (ISIS, Harwell, United Kingdom), O. Davies, and M. Tully (ESRF, FR) for helpful discussions and access to datasets. We acknowledge the SIBYLS beamline (Advanced Light Source, Berkeley, California) and B21 (Diamond Light Source) for data collection time. J.A.T. acknowledges support by 10.13039/100000002NIH R35 CA220430 and P30 GM124169, Robert A. Welch Chemistry Chair, and the 10.13039/100004917Cancer Prevention and Research Institute of Texas. The SIBYLS synchrotron beamline 12.3.1 and initial efforts by R.P.R. were supported by the 10.13039/100000015Department of Energy, Office of Basic Energy Sciences, Integrated Diffraction Analysis Technologies (IDAT) program.

## Author contributions

R.P.R. and J.A.T. contributed to the conception and design of the study as well as data analysis and interpretation. R.P.R. developed the theory and mathematical implementations for coding in JAVA. R.P.R. and J.A.T. wrote the manuscript.

## Declaration of interests

The authors declare no competing interests.

## Supporting material

Document S1. Figures S1–S9 and supporting materials and methods

Document S2. Article plus supporting material

## References

[1] Brosey C.A., Tainer J.A. (2019). Evolving SAXS versatility: solution X-ray scattering for macromolecular architecture, functional landscapes, and integrative structural biology. Curr. Opin. Struct. Biol..

[2] Rambo R.P., Tainer J.A. (2013). Super-resolution in solution X-ray scattering and its applications to structural systems biology. Annu. Rev. Biophys..

[3] Rambo R.P., Tainer J.A. (2010). Bridging the solution divide: comprehensive structural analyses of dynamic RNA, DNA, and protein assemblies by small-angle X-ray scattering. Curr. Opin. Struct. Biol..

[4] Moore P.B. (1980). Small-angle scattering. Information content and error analysis. J. Appl. Crystallogr..

[5] Shannon C.E. (1948). A Mathematical Theory of Communication. Bell System Technical Journal.

[6] Rambo R.P., Tainer J.A. (2013). Accurate assessment of mass, models and resolution by small-angle scattering. Nature.

[7] Shannon C.E. (1998). Communication In The Presence Of Noise. Proc. IEEE.

[8] Glatter O. (1977). A new method for the evaluation of small-angle scattering data. J. Appl. Crystallogr..

[9] Svergun D.I. (1992). Determination of the regularization parameter in indirect-transform methods using perceptual criteria. J. Appl. Crystallogr..

[10] Glatter O., Kratky O. (1982).

[11] Liu H., Zwart P.H. (2012). Determining pair distance distribution function from SAXS data using parametric functionals. J. Struct. Biol..

[12] Houdayer J., Poitevin F. (2017). Reduction of small-angle scattering profiles to finite sets of structural invariants. Acta Crystallogr. A Found. Adv..

[13] Kabanikhin S.I. (2008). Definitions and examples of inverse and ill-posed problems. J. Inverse Ill-Posed Probl..

[14] Hansen S. (2000). Bayesian estimation of hyperparameters for indirect Fourier transformation in small-angle scattering. J. Appl. Crystallogr..

[15] Kim S.-J., Koh K., Gorinevsky D. (2007). An Interior-Point Method for Large-Scale -Regularized Least Squares. IEEE J. Sel. Top. Signal Process..

[16] Rambo R.P., Tainer J.A. (2010). Improving small-angle X-ray scattering data for structural analyses of the RNA world. RNA.

[17] Mason A.C., Rambo R.P., Eichman B.F. (2014). A structure-specific nucleic acid-binding domain conserved among DNA repair proteins. Proc. Natl. Acad. Sci. USA.

[18] Classen S., Hura G.L., Tainer J.A. (2013). Implementation and performance of SIBYLS: a dual endstation small-angle X-ray scattering and macromolecular crystallography beamline at the Advanced Light Source. J. Appl. Crystallogr..

[19] Fasshauer G.E., Cheney W., Light W. (2004). A Course in Approximation Theory. Am. Math. Mon..

[20] Dunce J.M., Dunne O.M., Davies O.R. (2018). Structural basis of meiotic chromosome synapsis through SYCP1 self-assembly. Nat. Struct. Mol. Biol..

[21] Provost S.B. (1999).

[22] Akaike H. (1974). A new look at the statistical model identification. IEEE Trans. Automat. Contr..

[23] Bowerman S., Rana A.S.J.B., Wereszczynski J. (2017). Determining Atomistic SAXS Models of Tri-Ubiquitin Chains from Bayesian Analysis of Accelerated Molecular Dynamics Simulations. J. Chem. Theory Comput..

[24] Durbin J., Watson G.S. (1971). Testing for Serial Correlation in Least Squares Regression. III. Biometrika.

[25] Stoddard C.D., Montange R.K., Batey R.T. (2010). Free state conformational sampling of the SAM-I riboswitch aptamer domain. Structure.

[26] Byrne E.F.X., Sircar R., Siebold C. (2016). Structural basis of Smoothened regulation by its extracellular domains. Nature.

[27] Costantino D.A., Pfingsten J.S., Kieft J.S. (2008). tRNA-mRNA mimicry drives translation initiation from a viral IRES. Nat. Struct. Mol. Biol..

[28] Weiss T.M., Chaudhuri B., Muñoz I.G., Qian S., Urban V.S. (2017). Biological Small Angle Scattering: Techniques, Strategies and Tips.

[29] Pernot P., Round A., McSweeney S. (2013). Upgraded ESRF BM29 beamline for SAXS on macromolecules in solution. J. Synchrotron Radiat..

[30] Bizien T., Durand D., Pérez J. (2016). A Brief Survey of State-of-the-Art BioSAXS. Protein Pept. Lett..

[31] Pérez J., Vachette P., Chaudhuri B., Muñoz I.G., Qian S., Urban V.S. (2017). Biological Small Angle Scattering: Techniques, Strategies and Tips.

[32] Hura G.L., Menon A.L., Tainer J.A. (2009). Robust, high-throughput solution structural analyses by small angle X-ray scattering (SAXS). Nat. Methods.

[33] Rambo R.P., Tainer J.A. (2011). Characterizing flexible and intrinsically unstructured biological macromolecules by SAS using the Porod-Debye law. Biopolymers.

[34] Putnam C.D., Hammel M., Tainer J.A. (2007). X-ray solution scattering (SAXS) combined with crystallography and computation: defining accurate macromolecular structures, conformations and assemblies in solution. Q. Rev. Biophys..

[35] Grishaev A., Wu J., Bax A. (2005). Refinement of Multidomain Protein Structures by Combination of Solution Small-Angle X-ray Scattering and NMR Data. J. Am. Chem. Soc..

[36] Whitley M.J., Xi Z., Gronenborn A.M. (2017). A Combined NMR and SAXS Analysis of the Partially Folded Cataract-Associated V75D γD-Crystallin. Biophys. J..

[37] Thompson M.K., Ehlinger A.C., Chazin W.J. (2017). Analysis of functional dynamics of modular multidomain proteins by SAXS and NMR. Methods Enzymol.

[38] Conley M.J., McElwee M., Bhella D. (2019). Calicivirus VP2 forms a portal-like assembly following receptor engagement. Nature.

[39] Lai Y.-T., Hura G.L., Yeates T.O. (2016). Designing and defining dynamic protein cage nanoassemblies in solution. Sci. Adv..

[40] Brunette T.J., Parmeggiani F., Baker D. (2015). Exploring the repeat protein universe through computational protein design. Nature.

[41] Yan C., Dodd T., Ivanov I. (2019). Transcription preinitiation complex structure and dynamics provide insight into genetic diseases. Nat. Struct. Mol. Biol..

