# Supplementary material for: Information theory optimization of signals from small-angle scattering measurements
Source: Biophys J. 2025 Jun 27;124(15):2511–22. doi: 10.1016/j.bpj.2025.06.031 (PMC12414664; doi:10.1016/j.bpj.2025.06.031)
Supplement: Document S1. Figures S1–S9 and supporting materials and methods [file mmc1.pdf]

Biophysical Journal, Volume *124*

Supplemental information

Information theory optimization of signals from small-angle scattering measurements

Robert P. Rambo and John A. Tainer

1 Supporting Information

1.1 Presentation of the Moore sine integral transform method

SAXS of dilute, non-interacting homogeneous particles in solution is described by the Debye equation that relates, at a given q_j, the distribution of internal distances to an observed $I(q_j)$ through a sinc integral transform.

$$I(q_j) = 4\pi \int P(r)\,\frac{sin(q_j r)}{q_j r}\,dr \tag{1}$$

To put (1) in standard form, we rearrange terms q and r as

$$q_j \cdot I(q_j) = 4\pi \int \frac{P(r)}{r}\,sin(q_j r)\,dr \tag{2}$$

and by letting $Q(r) = P(r)/r$ and $H(q) = q \times I(q)$ and substituting yields

$$H(q_j) = 4\pi \int Q(r)\,sin(q_j r)\,dr \tag{3}$$

This clearly demonstrates that the relationship between real and reciprocal space is through a sine-integral transform. $Q(r)$ is an odd function since $Q(-r)$ must be equal to $-Q(r)$. $Q(r)$ can be represented by any orthogonal series expansion compatible with Legendre polynomials or odd functions such as the Fourier sine series and Chebyshev polynomials. Using the Fourier sine series, Moore showed the integral in (3) is a linear function with unknowns, a_{m}.

$$U(q_j) = 2\,d_{max}\sum_{m=1}^{N_s} a_m \cdot m \cdot (-1)^{m+1}\frac{sin(q_j d_{max})}{(m\pi)^2 - (q_j d_{max})^2} \tag{4}$$

In matrix form, $\vec{p}$ represent the Moore coefficients, a_{m}, for m in $\{1...N_{\mathrm{s}}\}$. The **S**-matrix will contain J rows (one row for each data point at q_j) and N_{s} columns. Each element of the **S**-matrix will contain a single term from the series calculated at a specified q_j and m value.

$$\begin{bmatrix} 1\cdot(-1)^{1+1}\frac{sin(q_1 d_{max})}{(1\cdot\pi)^2-(q_1 d_{max})^2} & \cdots & N_s\cdot(-1)^{N_s+1}\frac{sin(q_1 d_{max})}{(N_s\pi)^2-(q_1 d_{max})^2} \\ \vdots & \vdots & \vdots \\ \vdots & \vdots & \vdots \\ 1\cdot(-1)^{1+1}\frac{sin(q_J d_{max})}{(1\cdot\pi)^2-(q_J d_{max})^2} & \cdots & N_s\cdot(-1)^{N_s+1}\frac{sin(q_J d_{max})}{(N_s\pi)^2-(q_J d_{max})^2} \end{bmatrix} \cdot \begin{bmatrix} a_1 \\ \vdots \\ \vdots \\ a_{N_s} \end{bmatrix} = \begin{bmatrix} q_1 \cdot I(q_1) \\ \vdots \\ \vdots \\ q_J \cdot I(q_J) \end{bmatrix} \tag{5}$$

Therefore, the unknowns in $\vec{p}$ used in equation 7 can be recovered through an inversion of the **S**-matrix using a suitable pseudo-inverse method. In the presence of constant background, an additional column of ones and element will be added to the **S**-matrix and $\vec{p}$, respectively.

$$\begin{bmatrix} 1 & 1\cdot(-1)^{1+1}\frac{sin(q_1 d_{max})}{(1\cdot\pi)^2-(q_1 d_{max})^2} & \cdots & N_s\cdot(-1)^{N_s+1}\frac{sin(q_1 d_{max})}{(N_s\pi)^2-(q_1 d_{max})^2} \\ \vdots & \vdots & \vdots & \vdots \\ \vdots & \vdots & \vdots & \vdots \\ 1 & 1\cdot(-1)^{1+1}\frac{sin(q_J d_{max})}{(1\cdot\pi)^2-(q_J d_{max})^2} & \cdots & N_s\cdot(-1)^{N_s+1}\frac{sin(q_J d_{max})}{(N_s\pi)^2-(q_J d_{max})^2} \end{bmatrix} \cdot \begin{bmatrix} a_0 \\ a_1 \\ \vdots \\ a_{N_s} \end{bmatrix} = \begin{bmatrix} q_1\cdot I(q_1) \\ \vdots \\ \vdots \\ q_J\cdot I(q_J) \end{bmatrix} \tag{6}$$

The Moore coefficients, a_{m}, can be used to calculate the P(r)-distribution

$$P(r) = \frac{1}{2\pi^2}\cdot r\cdot\sum_{m=1}^{N_s} a_m\, sin\left(\frac{m\cdot\pi\cdot r}{d_{max}}\right) \tag{7}$$

1.2 Presentation of the Legendre orthogonal series expansion

Similar to the Moore method, the Legendre series expansion approximates the intensity data as $q\times I(q)$ with the exception that we are only expanding the unknown distribution, $P(r)$, and not $P(r)/r$. Here, the density function, $P(r)$, is strictly defined within the interval [-1,1] and can be approximated using a Legendre polynomial expansion as

$$P(r) = \sum_{m=0}^{\infty}\lambda_m\, L_m\left(\frac{2\cdot r - d_{max}}{d_{max}}\right) \tag{8}$$

The expansion is infinite but we will restrict the approximation to a fixed number of polynomials up to N_s. The evaluation of Legendre polynomial of order m, L_m, occurs at specified r-values that define the $P(r)$-distribution calculated as half-integer increments of the bin-width (b_{w})

$$b_w = \frac{d_{max}}{N_s} \Rightarrow r_i = (0.5+i)\cdot b_w\ where\ i\in\{0\cdots N_s\} \tag{9}$$

N_s is taken as the ceiling of the product of $(q_{max}\cdot d_{max}\cdot\pi^{-1})$. The matrix representation using the Legendre polynomials is given by

$$\begin{bmatrix} \sum\frac{sin(q_1 r)}{r}\cdot L_0\left(\frac{2\cdot r-d_{max}}{d_{max}}\right) & \cdots & \sum\frac{sin(q_1 r)}{r}\cdot L_{N_s}\left(\frac{2\cdot r-d_{max}}{d_{max}}\right) \\ \vdots & \vdots & \vdots \\ \vdots & \vdots & \vdots \\ \sum\frac{sin(q_J r)}{r}\cdot L_0\left(\frac{2\cdot r-d_{max}}{d_{max}}\right) & \cdots & \sum\frac{sin(q_J r)}{r}\cdot L_{N_s}\left(\frac{2\cdot r-d_{max}}{d_{max}}\right) \end{bmatrix} \cdot \begin{bmatrix} \lambda_0 \\ \lambda_1 \\ \vdots \\ \lambda_{N_s} \end{bmatrix} = \begin{bmatrix} q_1\cdot I(q_1) \\ \vdots \\ \vdots \\ q_J\cdot I(q_J) \end{bmatrix} \tag{10}$$

which shows the $\vec{p}$ contains the unknown Legendre coefficients and the **S**-matrix contains product-sum terms where each element is a sum over all r-values involving the product of a Legendre polynomial of fixed order and a sine term. The $\vec{p}$ can be recovered using a suitable matrix inversion technique or least squares.

1.3 Moore Method with Regularization

The Moore method determines a set of unknown coefficients that describe both the intensity data (4) and the real-space $P(r)$-distribution (7). We can use 4 in a regularization scheme where the canonical objective function in least squares is augmented (11) with the L2-norm of the second derivative of the $P(r)$-distribution (7).

$$minimize: \; \|S \cdot \vec{p} - U(\vec{q})\|^2 + \alpha \cdot \left\| \frac{d^2}{dr^2} P(r) \right\|_2 \tag{11}$$

The L2-norm of the second derivative acts as a smoothness constraint and is strictly positive. A perfectly smooth function will have a constant slope where the second derivative is zero.

$$\left\| \frac{d^2}{dr^2} P(r) \right\|_2 = \sum_{i=1}^{r\ values} \left(\frac{d^2}{dr^2} P(r_i) \right)^2 \tag{12}$$

Eqn. 7 is a closed-form, twice differentiable function that allows direct calculation of the second derivative at each r-value used in the $P(r)$-distribution (13).

$$\frac{d^2}{dr^2} P(r) = \frac{1}{\pi \cdot d_{max}} \sum_{m=1}^{N_s} a_m \cdot m \cdot cos\left(\frac{m \cdot \pi \cdot r}{d_{max}}\right) - r_i \cdot \frac{1}{\pi \cdot d_{max}^2} \sum_{m=1}^{N_s} a_m \cdot m^2 \cdot sin\left(\frac{m \cdot \pi \cdot r}{d_{max}}\right) \tag{13}$$

1.4 Propagation of Errors using SPI

Let $\mathbf{H}(q)$ be a data vector or Jx1 matrix with J rows (one row for each data point at q_j) whose elements $H(q_j) = q_j \cdot I(q_j)$. Each $I(q_j)$ has associated uncertainty (standard deviation) given by e_j therefore the scaled uncertainties for each $\mathbf{H}(q_j)$ is $e_j \cdot q_j$ forming the column vector $\overrightarrow{\delta}$ with same dimensions as $\mathbf{H}(q)$. We assume the noise associated with each element of $\mathbf{H}$ to be identical and independently distributed suggesting the associated covariance matrix to be an identity matrix. A complete description of our observation is given by

$$H(q_j) = h_j + e_j \cdot q_j = h_j + \delta_j \tag{14}$$

Singular value decomposition of the $\mathbf{S}$-matrix yields $\mathbf{S} = \mathbf{U\Sigma V}^T$ and we let $\mathbf{\Sigma}^+$ represent the inverse of the truncated singular value matrix restricted to the Shannon limit. $\mathbf{\Sigma}^+$ is a diagonal matrix where each element is the multiplicative inverse of $\mathbf{\Sigma}$ given by $1/\sigma_{\mathrm{i,i}}$. Therefore,

$$\mathbf{S}^+ = \mathbf{V\Sigma^+U^T} \tag{15}$$

The parameter vector, $\vec{p}$, will be given by $\mathbf{S}^+ \times \mathbf{H}(q)$

$$\mathbf{S}^{+}\mathbf{H}(q) \implies p_i = \frac{v_{i,i}}{\sigma_{i,i}} \cdot u_i^T \cdot \mathbf{H}(q) \; i \in \{1, ...N_s\} \tag{16}$$

Expanding $\mathbf{H}(q_j)$, we can see that the propagated error ϱ_i for p_i is given by the second term in .

$$\frac{v_{i,i}}{\sigma_{i,i}} \cdot u_i^T \cdot h_j + \frac{v_{i,i}}{\sigma_{i,i}} \cdot u_i^T \cdot \vec{\delta} \tag{17}$$

Based on the pseudo inverse method, the standard error in the estimated parameter is the sum of the observation errors weighted by the respective column of the left singular values (dot product of $u_i^T$and $\vec{\delta}$). This sum is multiplied by the ratio of the respective singular value to right singular value. For a fixed q-range, the singular values are determined by the structure of the **S**-matrix, a consequence of the Shannon limit, and not the quality of the data. It is clear that for a p_i determined by a small singular values ($\sigma_{i,i}$ < 1), the associated error may be enlarged unless the associated experimental errors are measured to a requisite accuracy. However, the dot product suggests a more useful insight where increasing the number of measurements between the fixed q-range can improve the error estimate for parameters with small singular values. A column from the U-matrix is orthonormal implying a unit magnitude and a dot product with u_i^T becomes a weighted average of $\vec{\delta}$. Assuming $\vec{\delta}$ to be a vector of randomly sampled errors, we can expect from the Law of Large numbers that the estimated parameter will converge to its expected value as we increase the number of observations within a fixed q-range. This suggests that detectors or camera geometries that increase the effective number of observations within a fixed q-range can improve the reliability of low magnitude singular values which coincide with the higher resolution information consistent with the Shannon-Hartley noisy-coding channel theorem.

1.5 Variances in Estimated Parameters via Cross-Validation

BioSAXS datasets are highly oversampled with the Shannon points occurring at defined points given by $q_n = n \cdot \pi / d_{max}$. An alternative method to estimate the variances in $\vec{p}$ can be devised through a repeated random sampling of points from the data matrix $\mathbf{H}(q)$ akin to a k-fold cross-validation scheme or Monte Carlo method. Here, the data is divided into bins where each q_n is separated by π/d_{max}. The Shannon points are mid-point to each respective bin. Data points are then sampled from each bin and used in the direct transform to estimate $\vec{p}$. Averaging and calculating the standard deviation of $\vec{p}$ over repeated random samplings with replacement can show how sensitive the estimated $\vec{p}$ is to variations in the data (see SI Fig. 9).

1.6 Scoring Function

Determining an acceptable $P(r)$-distribution from the SAXS dataset can not be achieved reliably through a simple, residuals-based χ^2-like metric alone. Such a statistic is prone to over-fitting and does not account for

the correlations that exist in a SAXS dataset. Often, the acceptability of a real-space transform is achieved by the user where the quality of the $P(r)$-distribution is subjectively evaluated for smoothness, negative values and oscillations. Such a set of expectations was quantified by Svergun (1992 reference) into a set of perceptual criteria and implemented in the program GNOM. In our implementation, we recognize that a search is initially performed over some finite d_{max} space. For d_{max} values that are too short, χ^2-like metrics tend to be good enough to recognize poorly fitting models (large residual discrepancies), however, when d_{max} values are too large, χ^2-like metrics become less discriminating as the increased model complexity produces small variations in the residuals. To compensate for this, we utilize the Akaike Information Criteria, AIC. AIC requires an estimate of the model's number of parameters, k, and likelihood score used to evaluate the model-data agreement. For low sample sizes, n, an additional term is required to give a sample size corrected $\mathrm{AIC_c}$ (Eqn. 13)

$$AIC = 2 \cdot k - 2 \cdot ln(L) + \frac{2k \cdot (k+1)}{n-k+1} \tag{18}$$

We can approximate the likelihood score, L, (Eqn. 19)using a residual-based metric such as χ^2.

$$L = \prod_{j}^{obs} \frac{1}{\sqrt{2\pi \cdot \sigma_j^2}}\, e^{-\frac{(I(q_j)_{calc} - I(q_j)_{obs})^2}{2\,\sigma_j^2}} \approx e^{-\chi^2} \tag{19}$$

To minimize the effects of the correlations in the SAXS dataset, we choose χ^2_{free} as our residuals-based metric. χ^2_{free} is normalized in our program therefore, we multiply by N_s to rescale. Finally, the number of parameters of the model, k, must include considerations regarding the choice of α, d_{max} and model (*i.e.*, Moore, SPI, or Legendre method), thus we define the total number of parameters, d_f, as $N_s + 3$. Finally, the total number of points or sample size used in the χ^2_{free} calculation is given by T. The AIC_c presented in the paper is given by Eqn. 20.

$$AIC_c = 2 \cdot d_f + N_s \cdot \chi^2_{free} + \frac{2d_f \cdot (d_f+1)}{T - d_f - 1} \tag{20}$$

To provide additional discriminating power to our score, we incorporate the Durbin-Watson (D_{W}) statistic (equation 21) which evaluates the randomness of the residuals calculated over all the data points, J, defined by $[q_{\min},\ q_{max}]$.

$$D_w = \frac{\sum_{j=2}^{J}(R_j \cdot R_{j-1})}{\sum_{j=1}^{J} R_j^2} \quad where \;\; R_j = q_j \cdot (I(q_j)^{calc} - I(q_j)^{exp}) \tag{21}$$

The D_{W} statistic is an autocorrelation calculated with a lag of 1. Ideally random residuals will have a D_{W} of 2 and is bounded between 0 and 4. Since we are seeking a minimum for our scoring function, we will use the absolute value of (2 - D_{W}) to inform on the quality of model-data agreement over the entire SAXS dataset defined by $[q_{\min},\ q_{max}]$.

Finally, to assess the quality of the $P(r)$-distribution, we quantify smoothness, presence of negative values

and oscillations. Smoothness is calculated as the totality of two different features: 1) the absolute sum of the second derivative calculated at the points that define the distribution function and 2) the finish near d_{max}. The second derivative sum is normalized to the integrated area of the distribution. To assess the finish at d_{max}, we recognize that $P(r)$-distributions calculated from atomistic coordinates are exceptionally shallow, the finish is free of bumps or bulges. Rather than calculating the slope, which will scale with the size of the particle or data normalization differences, we quantitate the finish as a ratio of two areas after the main peak of the $P(r)$-distribution. The $P(r)$-distribution consists of N points indexed from 0 to N-1 where $P(r_{N-1})$ is the value at d_{max}. We approximate the area of the distribution near d_{max} as a series of trapezoids starting at $P(r_{N-5})$. This area is subtracted from the area of a rectangle specified by the distance from r_{N-5} to d_{max} and height at $P(r_{N-5})$ and divided into the same rectangular area. Minimizing this area-based term, effectively seeks an area that minimizes bulges (see SI Fig 8).

$$smoothness \Rightarrow \frac{10}{\left|log_{10} \sum \frac{d^2}{dr^2} P(r)\right|} + 2 \cdot slope\_score \tag{22}$$

We use log_{10} the of the $P(r)$-distribution score since the sum of the second derivative can produce exceedingly small values. Consider second-derivative scores of 3.1x10^{-6} versus 7.8x10^{-7}, which will translate to 1.8 and 1.6 respectively using the first term in Eqn. 22.

To assess oscillations, we examine the bin heights for the points after the maximum of the $P(r)$-distribution. These points are examined at the Shannon limit, specifically at r-values defined by b_{w}. If there are N_s values in the distribution, indexed from $i = 0 \cdots (N-1)$, then $r_{N-1} = d_{max}$. We define an oscillation by noting that the last point before d_{max} or r_{N-2} should be less than the previous point, $P(r_{N-2}) < P(r_{N-3})$ and will always be greater than the point at r_{N-1} since $P(d_{max}) = 0$. Therefore, an oscillation will be seen as alternating heights at r-values indexed at odd integers, f in {3, 5, 7, ...}, where $P(r_{N-f}) < P(r_{N-f+1})$ and $P(r_{N-f}) < P(r_{N-f-1})$. The oscillations must persist through consecutive numbers within f.

Algorithm 1 Penalizing oscillations

$f \leftarrow 3$
$N \leftarrow total\ data\ points$
$total\_after\_peak \leftarrow N - index\_of\_peak$
$continue\_on \leftarrow true$
$penalty \leftarrow 0$
while $f < total\_after\_peak$ AND $continue\_on$ **do**
  if $P(r_{N-f}) < P(r_{N-f+1})$ AND $P(r_{N-f}) < P(r_{N-f-1})$ **then**
    $diff \leftarrow min(P(r_{N-f+1}) - P(r_{N-f}), P(r_{N-f-1}) - P(r_{N-f}))$
    $penalty \leftarrow penalty + 100 * diff/P(r_{N-f})$
    $f \leftarrow f + 2$
  else
    $continue\_on \leftarrow false$

The presence of negative values is scored by counting the number of $P(r)$ points, x, that are less than 0 but penalizing more severely if the last point before d_{max} is negative. We appreciate that some particles, such as detergent micelles, may demonstrate negative values within the body of the distribution and these are penalized least by only considering negative values after the peak of the distribution. This can be succinctly represented by a ternary expression in Eqn. 23.

$$penalty_{neg} = (P(r_{N-2}) < 0)\ ?\ (19.33 \cdot 7^{x})\ else\ (1.933 \cdot 7^{x}) \tag{23}$$

Our overall P(r)-distribution score that we seek to minimize is given by Eqn24.

$$Pr\_score = \left(\frac{10}{\left| log_{10} \sum \frac{d^2}{dr^2} P(r) \right|} + 2 \cdot slope\_score \right) + penalty_{osc} + penalty_{neg} \tag{24}$$

1.7 Implementation in Java

The SPI, Moore and Legendre methods are implemented in the JAVA program Scatter available at https://bl1231.als.lbl.gov/sc [illegible] and available on GITHUB at https://github.com/rambor/scatterIV. The SPI method with the L1-norm is based on a Matlab implementation by Boyd. Both the Moore and Legendre methods have been implemented with the L2-norm and make use of the Apache Math Commons library (https://commons.apache.org/proper/commons-math/). In all cases, setting α to zero solves the unregularized problem.

In the Scatter project, under the source directory src/version4/InverseTransform, will be the JAVA classes for the methods: SPI (class SVD), SPI with L1-norm (class SineIntegralTransform), Legendre (class LegendreTransform) and Moore (class MooreTransformApache). Implementation of Eqn. 24 can be found in the IndirectFT.java class under the method scoreDistribution.

2 Supporting Figure

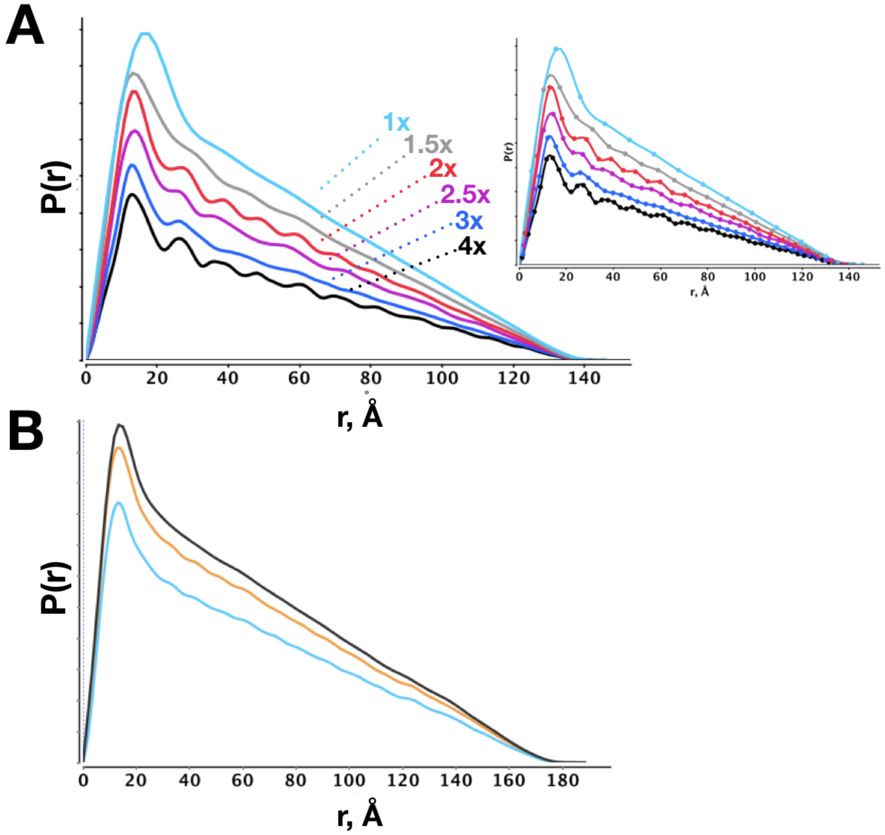

SI Fig. 1: P(r)-distributions calculated from crystal structures at decreasing bin-widths. A) 137 base-paired double-stranded DNA structure. Bin-widths are calculated at multiples of the B-form helix pitch at k·π/(10.4 Å) for k:{1, 1.5, 2, 2.5, 3, 4} representing q_{max} values of 0.302, 0.453, 0.604, 0.755, 0.906, and 1.208 Å^{-1}. Inset shows the actual points (bin heights) determined at each resolution. B) Coiled-coil protein (PDB 5XG2) calculated at q_{max} values of 0.4 (black), 0.6 (orange) and 0.8 (cyan) Å^{-1} representing relative increments of 1x, 1.5x and 2x. Increasing qmax shows oscillatory features that are directly derived from the structure. Curves are offset for illustrative purposes. DNA model was kindly provided by Nathan Cowieson, Diamond Light Source, Didcot,UK.

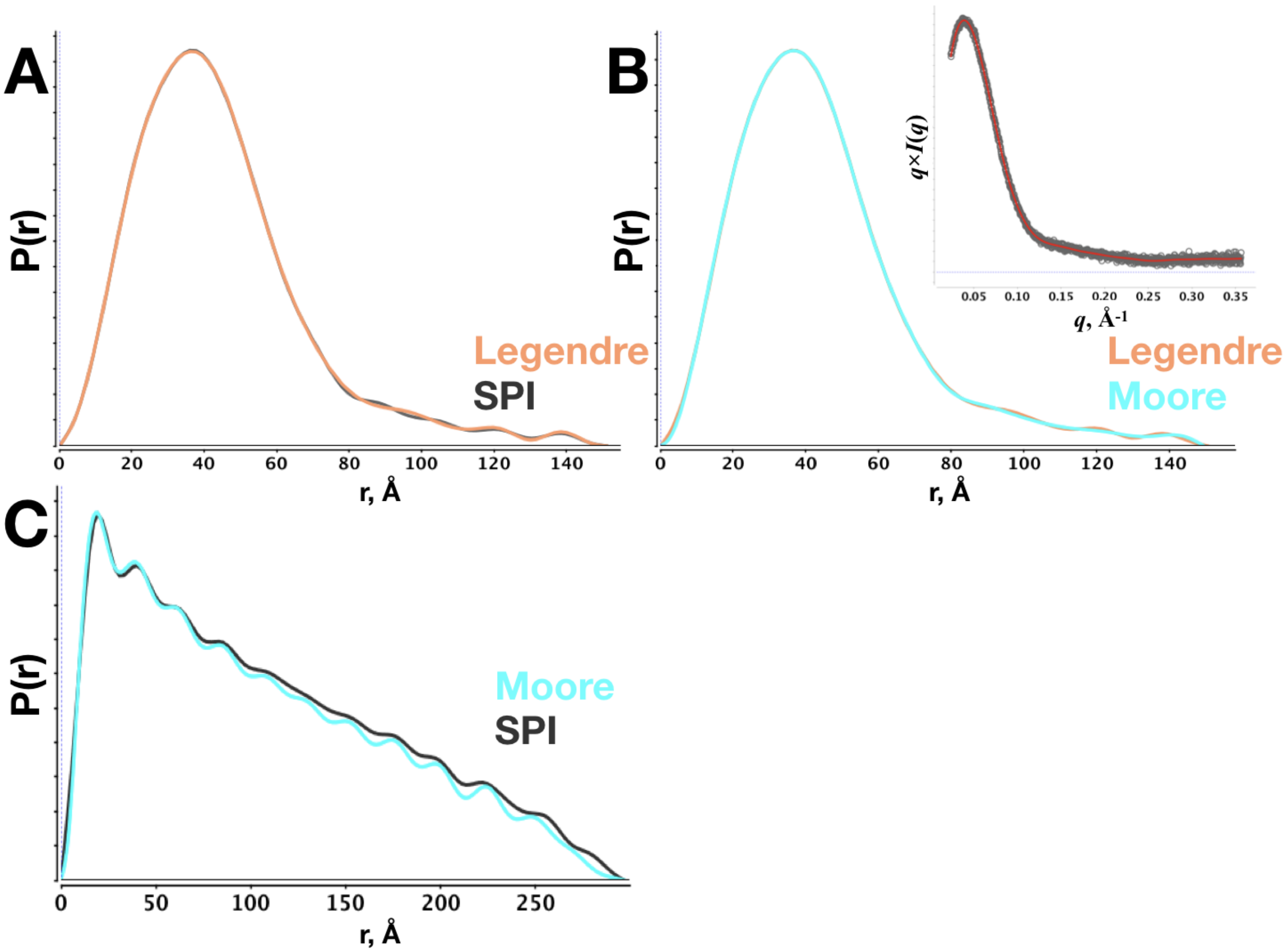

SI Fig. 2: Comparison of Legendre, Moore and SPI inverse transform methods without regularization ($\alpha = 0$). A) Overlay of the Legendre polynomial expansion (orange) on the SPI method (gray). B) Overlay of Moore method (cyan) on the Legendre polynomial expansion (orange). Inset shows the SAXS data (black circles) transformed as $q \times I(q)$ with the fit from the Moore method (red line). Data in A and B are un-purified BSA in PBS buffer at 3 mg per mL. C). Overlay of the Moore method (cyan) on the SPI method (gray) using SAXS data of a coiled-coil protein from Figure 2D.

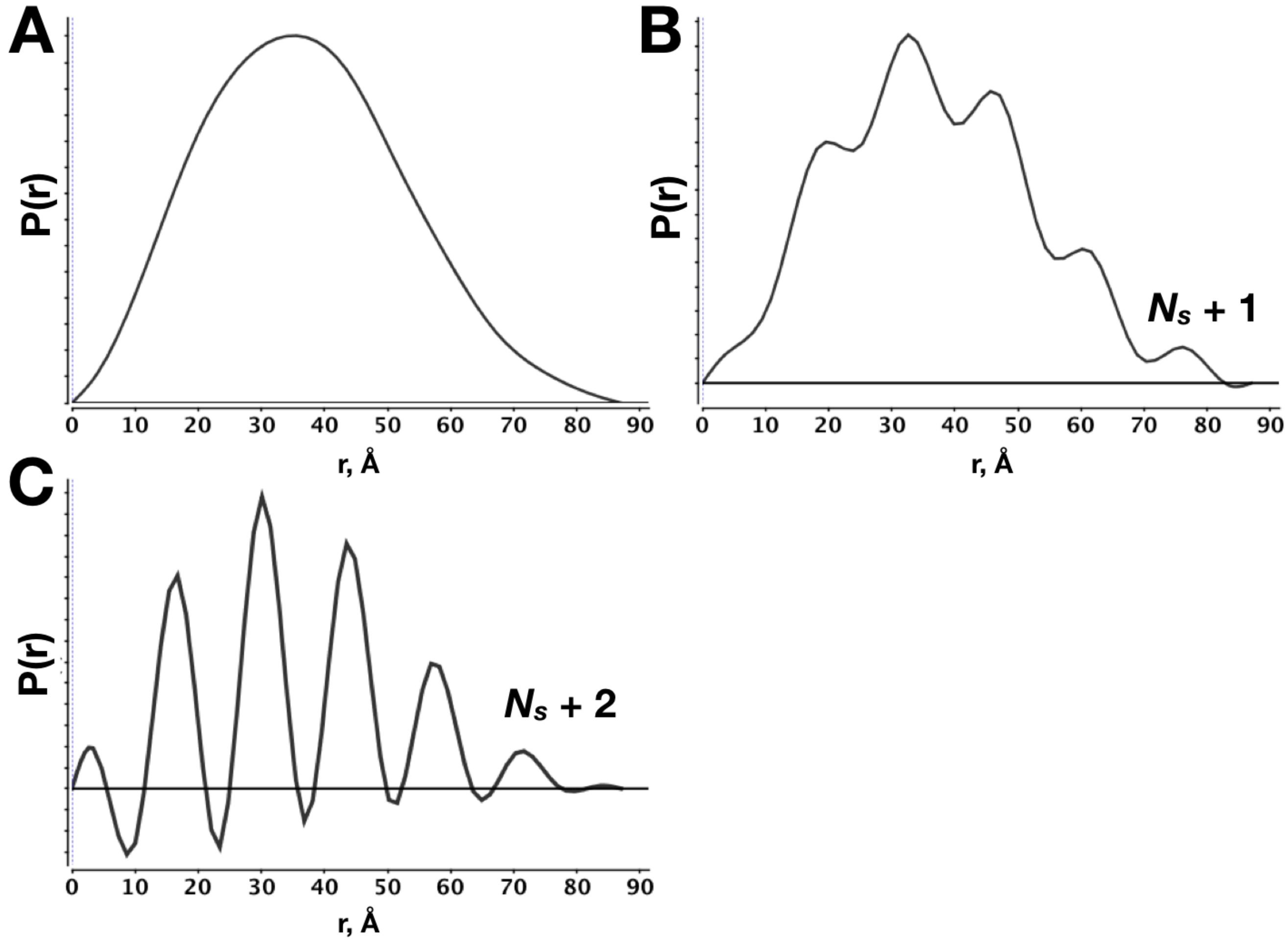

SI Fig. 3: Ill-conditioning of the inverse transform without regularization as the number of unknowns exceed N_s using the SPI method demonstrated with SEC-SAXS dataset of BSA from Figure 1. A) Transform calculated with N_s. B, C Transforms calculated with unknowns exceeding N_s.

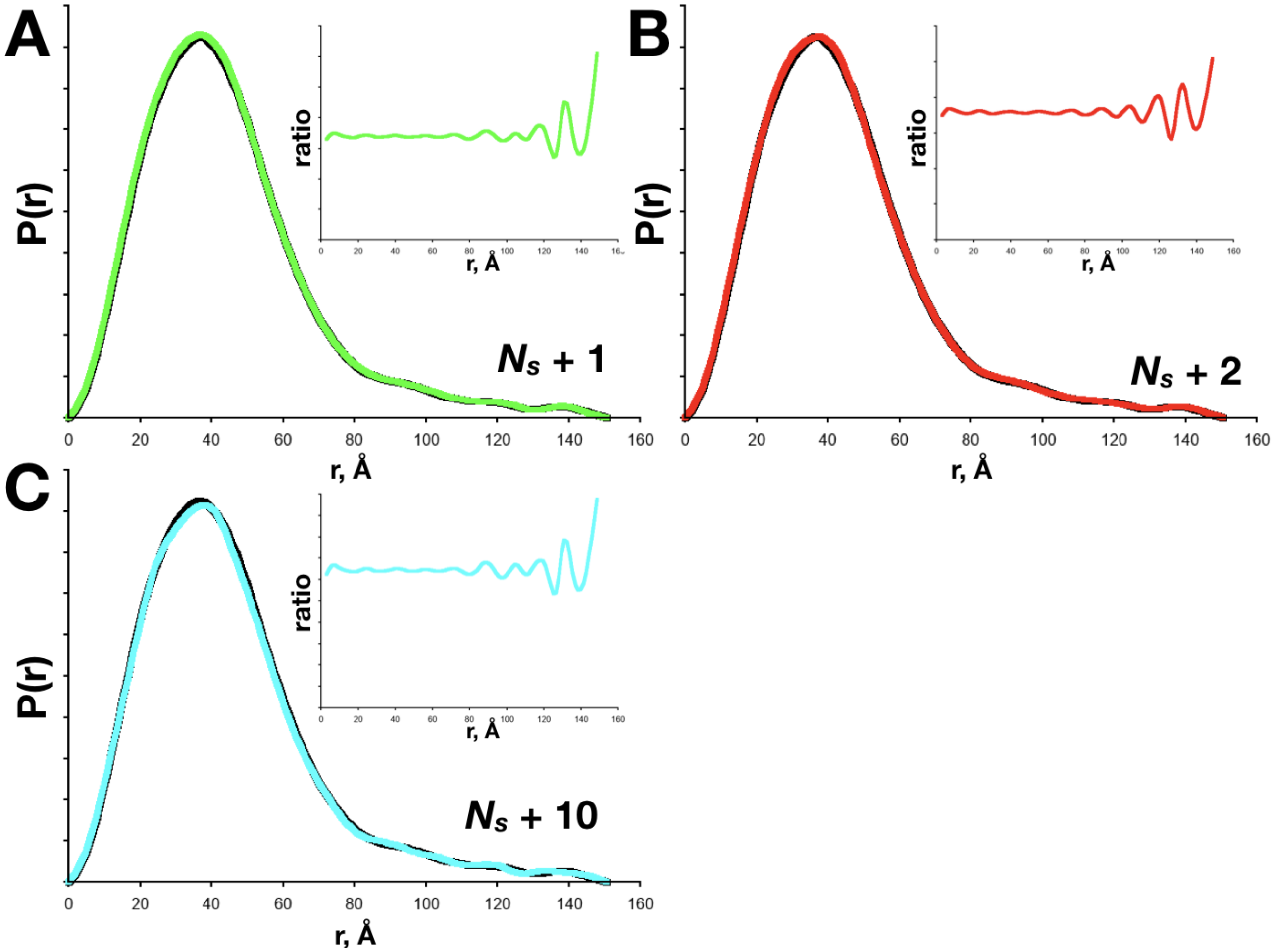

SI Fig. 4: Ill-conditioning of the inverse transform as the number of unknowns exceed N_{s} using an orthogonal series expansion (Legendre polynomials). SEC-SAXS dataset of BSA from Figure 1. Overlay of $P(r)$-distributions calculated using Shannon-limited terms at N_{s} (black), $N_{\mathrm{s}}+1$ (green), $N_{\mathrm{s}}+2$ (red) and $N_{\mathrm{s}}+10$ (cyan) at a fixed d_{max} of 150.5 without constant background. For each panel, inset illustrates the ratio of each ill-conditioned $P(r)$-distribution to the Shannon-limited $P(r)$-distribution. Undulations present in each ratio show that the distributions are fundamentally different.

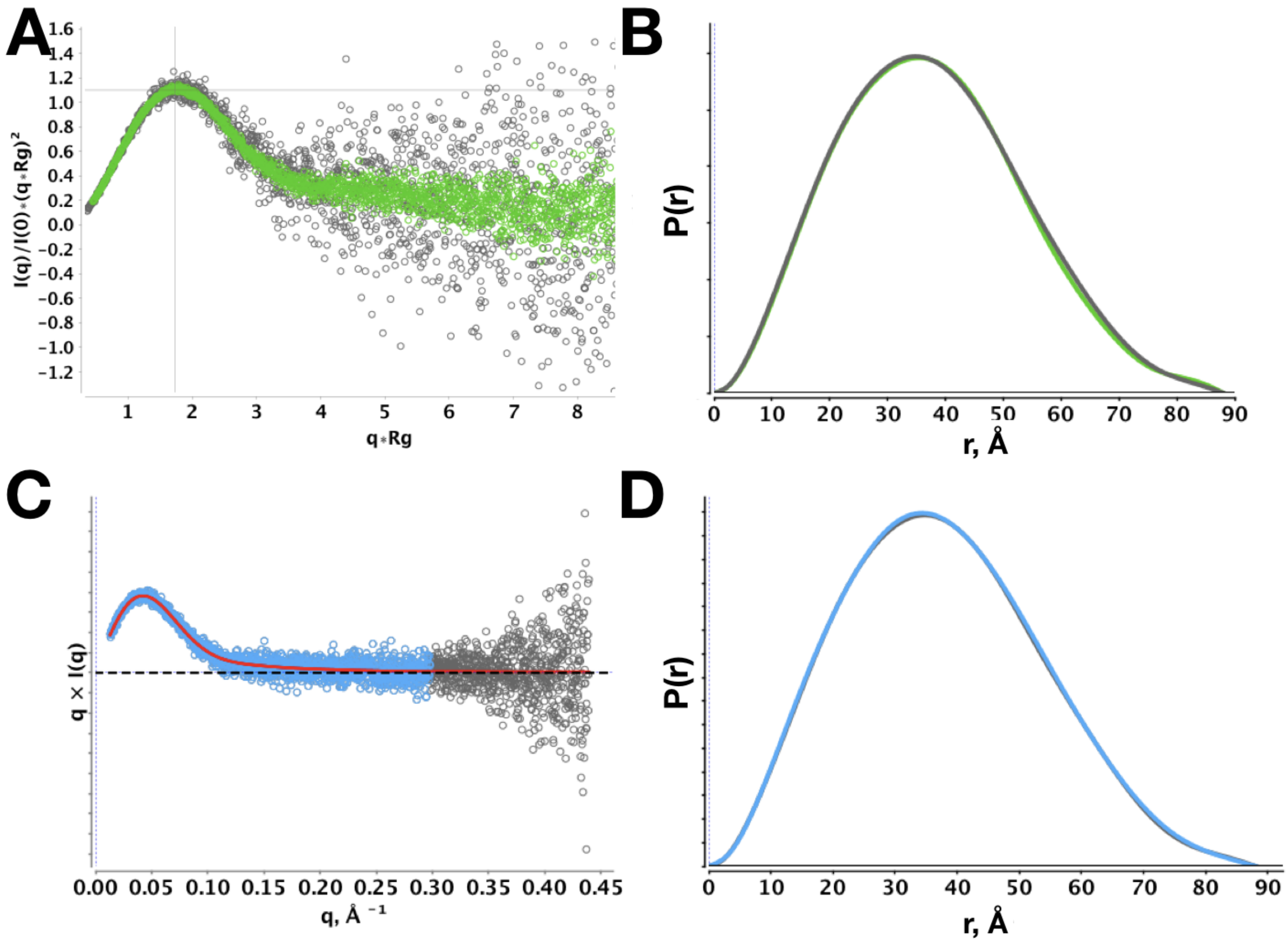

SI Fig. 5: Monomeric BSA from SEC-SAXS of dilute BSA in PBS buffer. A) Dimensionless Kratky plot of average of frames across a single elution peak (dark gray circles) corresponding to $\approx$ 0.26 mg per ml versus average of 18 independent (green), identical SEC-SAXS runs of monomeric BSA. B) Moore-method with smoothness regularization for both datasets in A. C) Average of frames across a single elution peak as in A but also truncated (blue circles) to $\approx$ 0.3 Å^{-1}. D) Inverse transform of data in C via Moore-method with smoothness regularization. For both B and D inverse transform was achieved with $\alpha = 331$, $d_{\max}$ 90.5 Å.

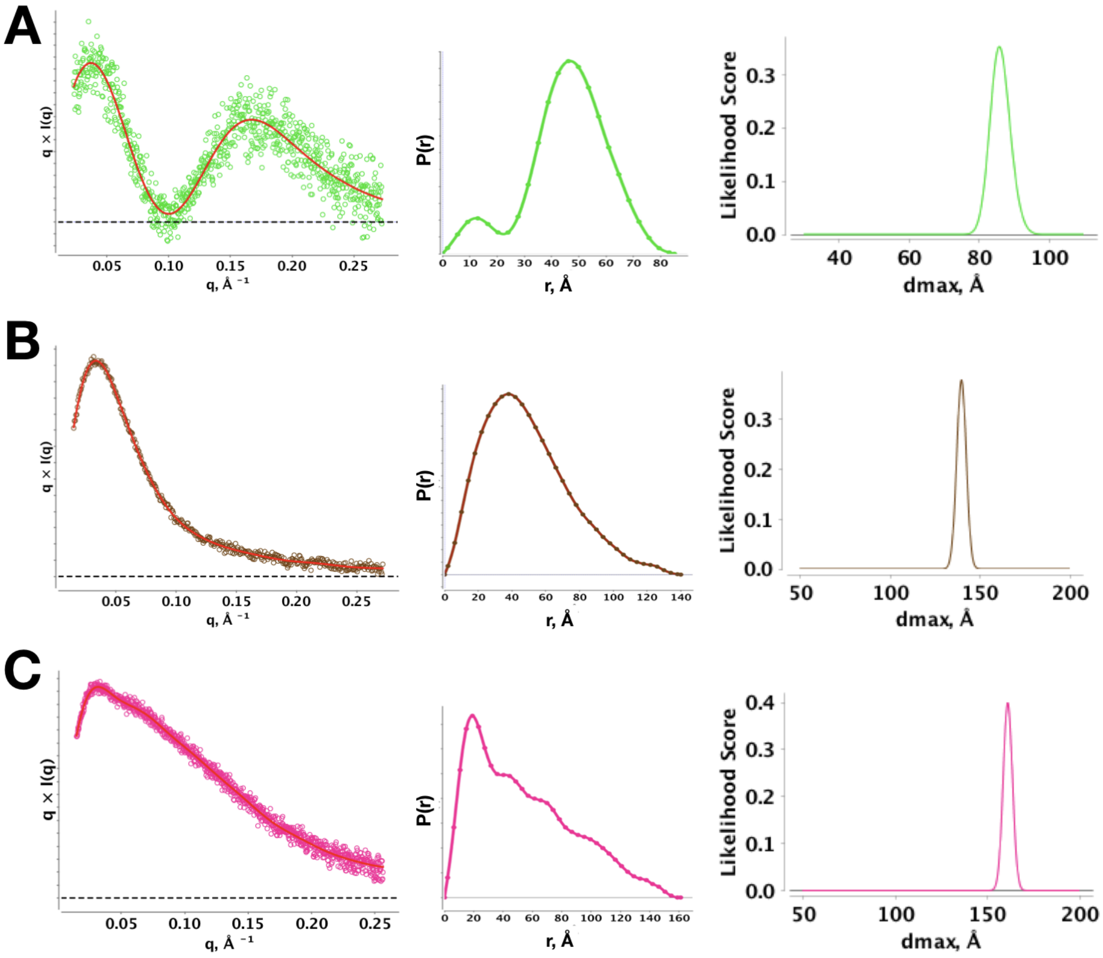

SI Fig. 6: Model selection using the Akaike information criteria based score. For each dataset, d_{max} searches were performed within a fixed range as specified by the x-axis of the plots on the right-hand side. The optimal $P(r)$-distribution is plotted along with the score-weighted averaged $P(r)$-distribution. The score-weighted averaged is calculated from the set of distributions contained within the peak(s) (red, central plots). Single best distribution is overlayed (colored circles). In some cases, it may be expected that the weighted averaged distribution is not coincident with the single best distribution. A) SEC-SAXS DDM micelle in PBS buffer. B) 25 base-paired, double-stranded DNA with 10 nucleotide single-stranded overhang from C) SEC-SAXS purified 50 base-paired double-stranded DNA in PBS.

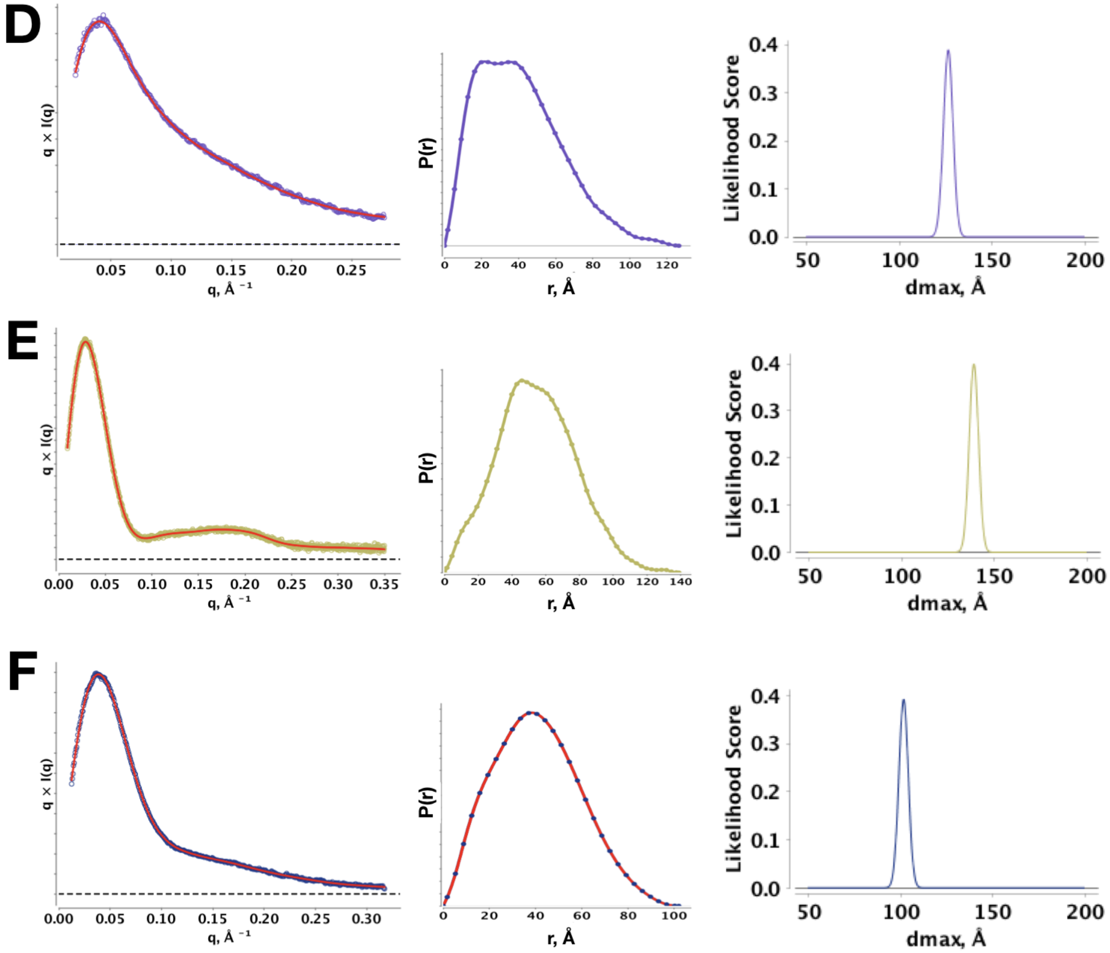

SI Fig. 7: Continue of SI Fig. 6 D) unfolded SAM-riboswitch RNA in presence of EDTA. E) SEC-SAXS of amphipol G-protein coupled receptor from. F) SEC-purified BMV RNA from Kieft.

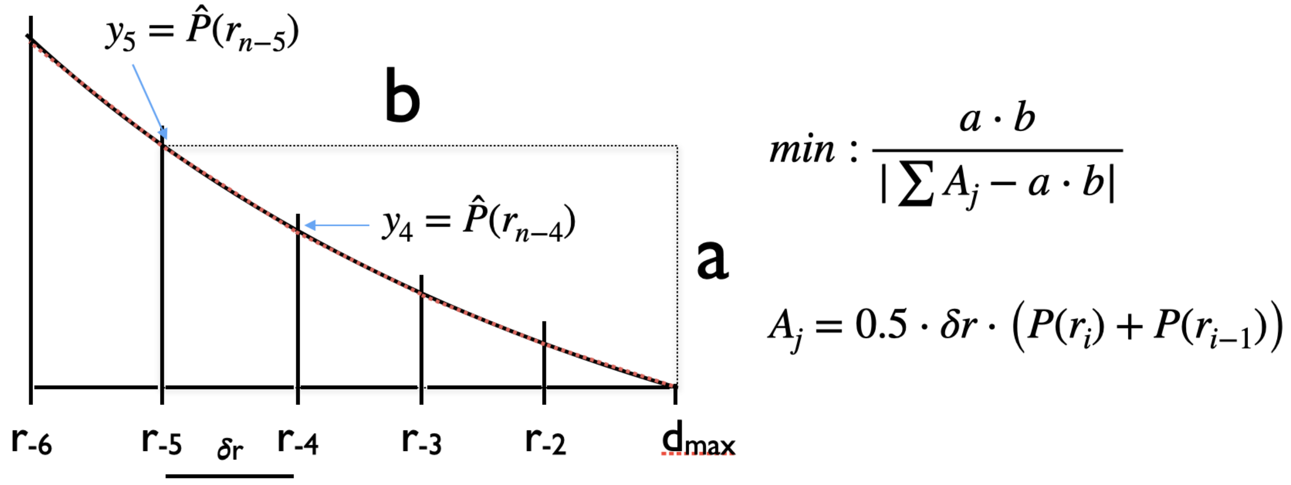

SI Fig. 8: Assessing the finish at d_{max}. The area near d_{max} is approximated as a series of trapezoids starting from 5th point from the end. This area is subsequently subtracted from the area of a rectangle defined by the sides '**a**' and '**b**'. The minimization target is the ratio of the rectangular area to the difference area. The difference area is the area outside of the distribution and minimization forces this area to be as large as possible.

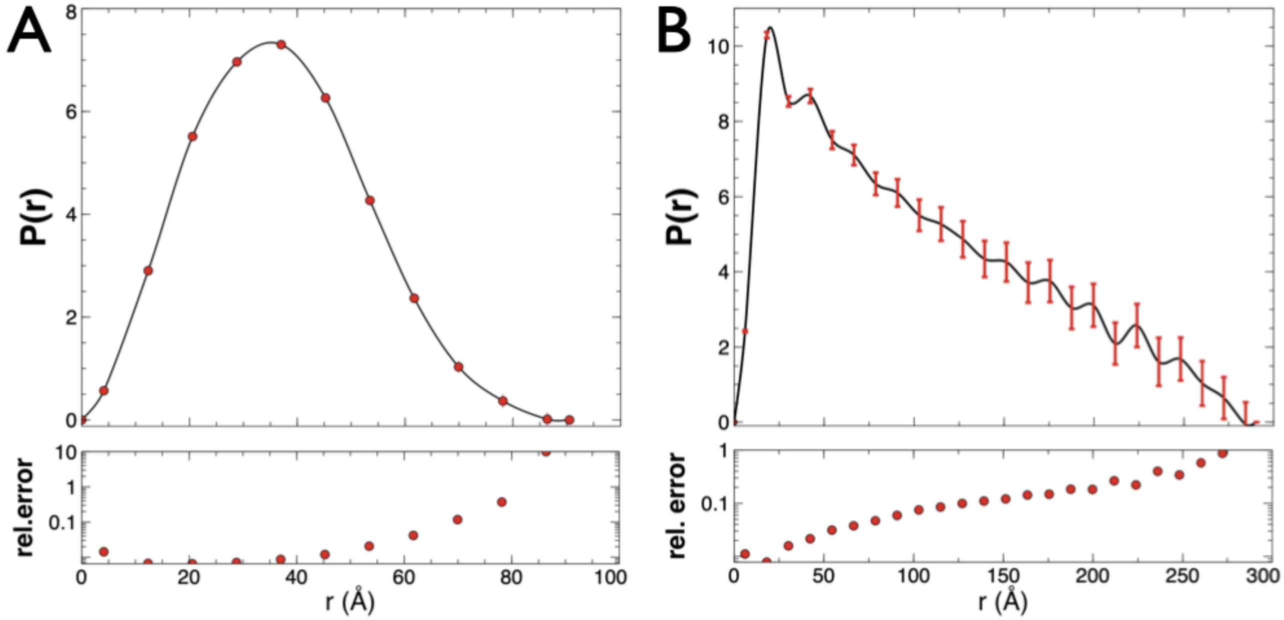

SI Fig. 9: Parameter error estimates for SPI without regularization using random sub-samplings. To estimate the uncertainty associated with the estimate parameters ($\vec{p}$), a SAXS dataset is divided into bins based on the Shannon limit, N_s. Each bin has a width π/d_{max} and centered at $q_n = n \cdot \pi/d_{max}$ where n is an integer from 1 to N_s. Approximately 13% of the SAXS data is randomly sampled from each bin to form a working set which is subsequently used in a direct inverse transform. The recovered $\vec{p}$ is averaged over 155 independent sampling rounds with replacement. Averaged $\vec{p}$ (red circles) with associated errors bars are plotted above with relative errors below (note log10 scale). **A**. BSA SAXS data from Figure 1, working set consisted of 158 data points drawn from a total of 1200 observations. **B**. KASH5 coil-coiled SAXS data from Figure 2, working set consisted of 142 data points drawn from a total of 1106 observations. Figures were prepared with Veusz (https://veusz.github.io).
